# Water Splitting and Transport of Ions in Electromembrane System with Bilayer Ion-Exchange Membrane

**DOI:** 10.3390/membranes10110346

**Published:** 2020-11-16

**Authors:** Stanislav Melnikov, Denis Bondarev, Elena Nosova, Ekaterina Melnikova, Victor Zabolotskiy

**Affiliations:** Kuban State University, Stavropolskaya 149, 350040 Krasnodar, Russia; bondarew.denis1992@gmail.com (D.B.); firofran@mail.ru (E.N.); ekaterinabelashova23@gmail.com (E.M.); vizab@chem.kubsu.ru (V.Z.)

**Keywords:** bilayer membrane, water splitting, ion transport, electrochemical impedance, current-voltage characteristic, ion transport numbers

## Abstract

Bilayer ion-exchange membranes are mainly used for separating single and multiply charged ions. It is well known that in membranes in which the layers have different charges of the ionogenic groups of the matrix, the limiting current decreases, and the water splitting reaction accelerates in comparison with monolayer (isotropic) ion-exchange membranes. We study samples of bilayer ion-exchange membranes with very thin cation-exchange layers deposited on an anion-exchange membrane-substrate in this work. It was revealed that in bilayer membranes, the limiting current’s value is determined by the properties of a thin surface film (modifying layer). A linear regularity of the dependence of the non-equilibrium effective rate constant of the water-splitting reaction on the resistance of the bipolar region, which is valid for both bilayer and bipolar membranes, has been revealed. It is shown that the introduction of the catalyst significantly reduces the water-splitting voltage, but reduces the selectivity of the membrane. It is possible to regulate the fluxes of salt ions and water splitting products (hydrogen and hydroxyl ions) by changing the current density. Such an ability makes it possible to conduct a controlled process of desalting electrolytes with simultaneous pH adjustment.

## 1. Introduction

Ion-exchange membranes are nanoporous polymeric materials, the functional properties of which are determined by the laws governing the transport of ions and water in pores with charged walls. Ion-exchange membranes have a large number of applications in the processes of purification, concentration and separation of substances [1,2], as well as synthesis processes [3,4,5,6]. The areas of application of membrane processes are steadily expanding. One of the new vectors for the development of electromembrane technologies is the processing of ampholyte-containing solutions [7,8,9] and the recovery of nutrients from the waste products of humans and animals [10,11].

Ampholytes are substances capable of attaching and donating protons. As a rule, these are acidic residues of weak inorganic (phosphoric, carbonic) or polybasic organic acids containing one or more protons attached to the acid group. Such ions can change their chemical form and/or charge as a result of a change in the pH of the solution, which means that the composition of the solution will change during the electrodialysis process. As a result, the transfer of ions and the properties of ion-exchange membranes in ampholyte solutions differ significantly from those in electrolyte solutions incapable of protonation/deprotonation. The transfer of ampholyte ions in electromembrane systems is complicated by the occurrence of chemical reactions, both in solution and in the membrane itself [12,13]. The interaction of organic ions with the membrane matrix can lead to a significant increase in the electrical resistance of the membrane [14], fouling of organic substances to the membrane surface [15], and general degradation of the membrane material associated with the need to clean the membrane from organic substances [16].

The currently produced ion-exchange membranes are either too expensive or created for the processing of strong electrolytes, and their characteristics do not meet the new requirements. The solution to these problems can be the creation of anisotropic ion-exchange membranes by applying modifying layers to the surface of the substrate membrane, that is, by creating bilayer and multilayer ion-exchange membranes.

Anisotropic membranes are most widely used in the field of baromembrane processes, where the selective and transport properties of reverse osmosis and nanofiltration membranes are determined by a thin active layer on the surface of a porous substrate [17,18]. Such membranes are anisotropic in the direction perpendicular to the membrane surface (the direction coincides with the direction of the substance flow through the membrane).

In the field of membrane electrochemistry, anisotropic membranes were first mentioned in the works of Frilette (bipolar membrane) [19], Leitz (as cationic-anionic) [20]. Both works describe an ion-exchange membrane consisting of cation-exchange and anion-exchange layers; the difference is that Leitz proposed a membrane which is capable to carry out the process of desalting of sodium chloride with simultaneous alkalization or acidification. To achieve this, the thickness of the anion layer of the bilayer membrane is one-half to one-quarter the thickness of the cation layer [20]. Here, we should note that the idea of combining the two processes in one electromembrane apparatus was proposed even earlier by Kollsman [21].

Subsequently, the idea of creating a membrane capable of simultaneously combining two mechanisms (desalting and changing the pH of the solution) was developed in the works of Antonov et al. [22] and Shendrik et al. [23]. The authors of [22,23] obtained membranes with a thin layer of microdispersed ion exchanger electrodeposited from solution onto the surface of the substrate membrane.

The authors of [22] called membranes capable of simultaneous desalting and shifting the pH of a solution “semi-bipolar”. The disadvantages of the obtained semi-bipolar membranes include the complete absence of mechanical stability and destruction of the electrodeposited layer when the electric field is turned off. Moreover, similar “semi-bipolar” membranes include membranes on the surface of which surfactants deposit (proteins, peptides, amino acids, etc.). Such deposition results in inversion of the membrane surface charge [24,25,26,27], thus creating a bilayer structure with layers with opposite charges.

It is possible to obtain mechanically stable “semi-bipolar” membranes if a modifying layer is made from an ion-polymer film. The authors of works [28,29,30] successfully solved a similar problem. The authors of these works used the term “asymmetric bipolar membranes” to describe the obtained anisotropic membranes. In these studies, to create a modifying layer, the casting of a solution containing an ionpolymer in a volatile solvent onto the membrane surface was used. At present, in addition to the casting method, there are several new technologies that allow creating thin layers on the surface of the substrate membrane: layer-by-layer deposition [31,32,33,34,35], electrostatic bonding [36,37,38], grafting [39,40,41], chemical bonding [42,43,44,45], electrospinning [46].

The deposition of thin layers/films on the surface of the substrate membrane is of the greatest importance for imparting specific selectivity to the membrane, as a rule, to singly charged ions. The results of studying such bilayer and multilayer membranes are presented in a large number of works and are summarized in recent reviews [47,48]. The focus of such works, as a rule, is the permselectivity coefficient, while other electrochemical characteristics of membranes are not studied. At the same time, in some works, researchers draw attention to a decrease in the value of the limiting current on the modified membrane, as compared to the original one [35,49,50].

According to the published reports, the modification materials for cation-exchange substrates are mainly focused on the some polyelectrolytes, such as polyethyleneimine (PEI), polyaniline, polypyrrole, polyallylamine, chitosan etc. [51].

In the case when the charge of the matrix of the modifying layer is opposite to the charge of the matrix of the membrane-substrate at the point of contact of these layers, a space charge region is formed, in which the electric field strength can reach values of the order of 10^9^ V/m. In the scientific literature on bipolar membranes, this area is commonly referred to as the “bipolar border” or “bipolar region”. Essentially, we can deduce that almost all of the reported membranes with surface modification are asymmetric bipolar membranes. Zabolotskiy et al. [29] had shown that asymmetric bipolar membranes can maintain high salt flux at low currents, while their function is similar to bipolar membranes at high current densities.

Conventional bipolar membranes are bilayer composites in which layers (cation- and anion-exchange) have ion selective properties. Such membranes can produce protons and hydroxyl ions from water molecules, under the influence of an imposed electric current. Such properties allow for creating a series of unique electromembrane processes using bipolar membranes, for example, adjusting the pH of solutions without the addition of chemicals and the formation of by-products or wastes, the conversion of salt solutions to acids and bases, the separation of ions, and the continuous electrochemical regeneration of ion exchangers in the production of ultrapure water [6,52,53].

Unlike acid and alkali production, pH adjustment does not require bipolar membranes with high selectivity. Moreover, it is convenient to combine the desalination process with a simultaneous pH adjustment. However, the flux of salt ions through the bipolar membrane is very low [54]. This circumstance limits the use of bipolar membranes in the process of desalting with the simultaneous adjustment of the pH of the solution. At the same time, the asymmetric bipolar membranes suit this niche very well.

The key features that determine the possibility of efficient use of bipolar membranes are the overall potential drop on the membrane and the current efficiency, both depending on the water splitting reaction kinetics. Improvement of the electrochemical characteristics of a bipolar membrane can be achieved by the introduction of a water splitting catalyst into the bipolar boundary. Introduction of the catalyst reduces the overvoltage of the bipolar region and, as a result, overall potential drop of the bipolar membrane. Such catalysts can be both organic [55,56,57,58,59,60,61] and inorganic [53,62,63,64,65,66,67,68] in nature.

In our previous works, we managed to achieve some understanding of the laws governing the processes of ion transfer in bilayer membranes. In [69], we showed the possibility of synthesizing inorganic catalysts for the water splitting reaction in an asymmetric bipolar membrane. In [29], we showed that the series-connected Gerischer and finite Warburg elements could describe an asymmetric bipolar membrane’s impedance spectrum. This nature of the membrane impedance indicated the presence of electrodiffusion transfer of salt ions through the asymmetric bipolar membrane, even in the presence of generation of hydrogen and hydroxyl ions. In [30,70], we showed that an ionpolymer catalyst for the reaction of water splitting in the cation-exchange layer of an asymmetric bipolar membrane leads to a significant decrease in the potential drop across the membrane. In [71], a mathematical model of the current-voltage characteristic of an asymmetric bipolar membrane was developed, which allows one to predict the dependence of the limiting current on the membrane on the thickness of the cation-exchange layer.

In the present work, we define asymmetric bipolar membranes and permselective membranes as bilayer membranes. We specifically refer to them as “bilayer membranes” to emphasize that their main function is not predetermined at the stage of synthesis but can change depending on operational conditions. We will show that the transport of salt ions and water splitting products through a bilayer membrane is complex and is affected by current density, nature and concentration of the solution, composition of the modifying layer. To do that, we will study the electrochemical characteristics of bilayer membranes in which a heterogeneous ion-exchange membrane was used as the substrate, both containing and not containing a water splitting catalyst under different working conditions.

## 2. Experimental

### 2.1. Objects of the Study

The objects of study in this work were bilayer membranes consisting of a thick anion-exchange layer (AEL) (a commercial membrane Ralex AMH-PES (produced by Mega a.s., Czech Republic) was used as an anion-exchange layer) and a thin cation-exchange layer (CEL). For research purposes, the thickness and chemical nature of the cation exchange layer were different. In addition, various additives were introduced into the cation-exchange layer to act as catalysts for the water splitting reaction. The size, chemical nature, and method of introduction of these additives also differed in different experiments.

The electrochemical characteristics of a bilayer membrane with a thick cation-exchange layer (a commercial Ralex CM-PES membrane) and a thin anion-exchange layer were studied separately. The anion exchange layer was made of a copolymer of *N*, *N*-dimethyl-*N*, *N*-diallylammonium chloride and ethyl methacrylate. The thickness of the anion-exchange layer, in this case, was constant at 80 μm.

Conventionally, all the studied membranes can be divided into two categories—membranes with a thin layer without a catalyst (named as BM-a series) and membranes with a catalyst for the water splitting reaction (BM-ac series).

Table 1 presents the acronyms and characteristics of the studied membranes.

Methods for membranes and water splitting catalysts preparation are presented in detail below.

#### 2.1.1. Preparation of Bilayer Membranes with a Thin Cation-Exchange Film (BM-a)

Commercial heterogeneous anion-exchange membrane Ralex AMH-PES (produced by Mega a.s., Straz pod Ralskem, Czech Republic) was used as an anion-exchange layer. The BM-a membranes were obtained by casting of a 5% solution of a sulfonated polytetrafluoroethylene in a solvent on the heterogeneous anion-exchange membrane-substrate, followed by evaporation of the solvent at 40 °C for 6 h. The casting solution was prepared by mixing of a 10% vol. of a sulfonated polytetrafluoroethylene in isopropyl alcohol and pure acetic acid in 1:1 volume ratio.

The casting solution composition was developed previously [71]. The used composition provides the best mechanical properties of the resulting membrane, while maintaining the maximum volume fraction of the sulfonated polytetrafluoroethylene.

To produce membranes with a thin sulfonated poly (ether ether) ketone (SPEEK) layer the following procedure was used: 4 g of granulated poly (ether ether) ketone was placed in 200 mL of concentrated sulfuric acid, and then left for 120 h at room temperature and intensive stirring (poly (ether ether) ketone granules were completely dissolved in sulfuric acid). After this time, the resulting solution was slowly poured into 500 mL of ice water, while the resulting polymer precipitated, which was filtered off under vacuum and washed with distilled water until the filtrate was neutral. The filtered polymer was dried in a vacuum desiccator.

The resulting polymer powder was dissolved in anhydrous dimethylformamide to obtain a 10% by weight solution. Then, acetic acid was added to the resulting solution in a volume ratio of 1:1 to obtain a casting solution. The resulting SPEEK solution was applied to the surface of the substrate membrane in an amount enough to form a cation-exchange layer 30 ± 2 μm thick.

To obtain a thin cation-exchange layer of sulfonated polystyrene (SPS), a sample of SPS weighing 8.9 g was dissolved in 100 mL of water. The resulting solution was also mixed with acetic acid in a 1:1 volume ratio. The resulting casting solution was applied to the membrane in an amount enough to obtain a membrane with a cation-exchange layer thickness of 30 ± 2 μm.

The thickness of the cation-exchange layer was determined as the difference between the measured thickness of the bilayer membrane in the swollen state and the thickness of the substrate membrane. A high-precision micrometer Mitutoyo Absolute Digimatic Quick 0–15/0.001 mm 227–201 (LLC “Mitutoyo Rus” Moscow, Russia) was used for measurements. The measurement error was ± 2 μm.

#### 2.1.2. Preparation of Bilayer Membranes with a Thin Anion-Exchange Film (BM-c-A80)

A membrane with a cation-exchange membrane-substrate was developed to study the properties of a bilayer membrane with a thin anion-exchange layer. The commercial membrane Ralex CM-Pes (produced by Mega a.s., Straz pod Ralskem, Czech Republic) was chosen as a cation-exchange layer. On the surface of a Ralex CM-PES cation-exchange membrane, a sulfonated polytetrafluoroethylene film with a thickness of 30 micrometers was formed in accordance with the procedure described above. Next, on the side of the membrane with a layer of sulfonated polytetrafluoroethylene, a 5% solution of a copolymer of *N*, *N*-dimethyl-*N*, *N*-diallylammonium chloride and ethyl methacrylate in acetone was applied in an amount required to obtain a layer of copolymer 80 μm thick, and left at room temperature for 24 h until the solvent has completely evaporated.

The thickness of the anion-exchange layer was determined in the same way as the thickness of the cation-exchange layer on the BM-a series membranes.

#### 2.1.3. Bilayer Membranes with Water Splitting Catalysts (BM-ac)

To obtain membranes active in the water splitting reaction, a suspension of catalyst particles in the casting solution was applied to the surface of the membrane-substrate. In all these samples, the sulfonated polytetrafluoroethylene with thickness of 30 microns was used as the CEL.

A powder of phosphoric cation-exchanger KF-1 was used as a catalyst. The mean size of the powder particles was 10 ± 2 μm; a small fraction (not more than 5%) of bigger particles up to 100 μm was also present in the powder.

The cation-exchange resin KF-1 was chosen as an additive to the cation-exchange layer, since previous studies have shown its high activity as a catalyst for the water splitting reaction [61]. To obtain microdispersion, spherical granules of the resin were ground in an SVM-04 vibration mill (balls diameter 8 mm, number of oscillations 25 s^−1^, engine power 0.37 kWh) for 20 min.

Iron (III) and chromium (III) hydroxides were also chosen as highly efficient catalysts for the water splitting reaction [62,69,72,73].

The microdispersion of transition metal hydroxides was obtained in several stages. Initially, precipitates of iron (III) and chromium (III) hydroxide were obtained by chemical precipitation from 0.5 M solutions of metal chlorides:

$${\mathrm{FeCl}}_{3}+3\mathrm{NaOH}\to \mathrm{Fe}{\left(\mathrm{OH}\right)}_{3}↓+3\mathrm{NaCl}\;{\mathrm{CrCl}}_{3}+3\mathrm{NaOH}\to \mathrm{Cr}{\left(\mathrm{OH}\right)}_{3}↓+3\mathrm{NaCl}$$

Then, the resulting precipitate was filtered and washed with distilled water to pH ≈ 7 using universal indicator paper. Then, the precipitate was dried and grounded in a ball mill under the same conditions as the powder of the KF-1 cation-exchanger.

The obtained microdispersed powders of catalysts were fractionated on a Fritsch Analysette 3 shaker to determine the fractional composition ( Supplementary Figure S1).

In the case of KF-1, the amount of powder applied to the membrane surface was varied. In the case of iron (III) and chromium (III) hydroxides, their amount was constant and amounted to 2 mg/cm^2^.

#### 2.1.4. Electrodeposition of Transition Metal Hydroxides on the Bipolar Boundary

**Figure 1:**
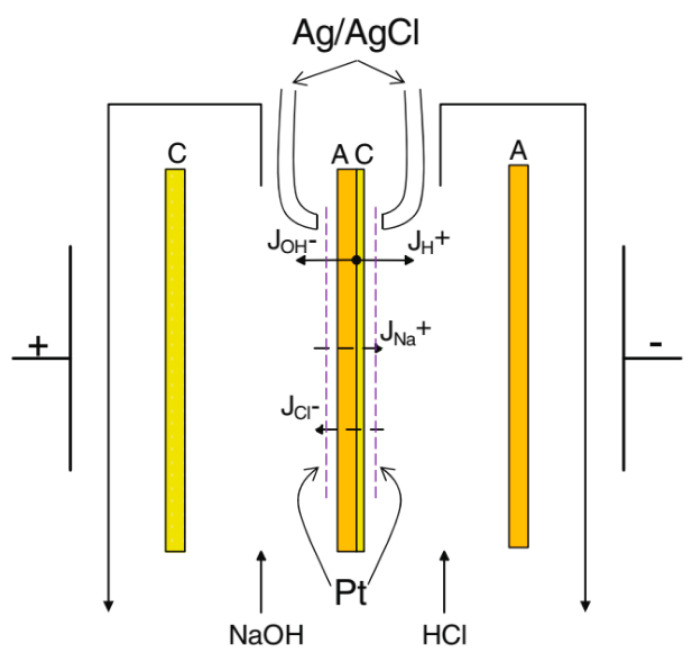
The scheme of the experimental cell and ion fluxes through the studied membrane. C—cation-exchange membrane; A—anion-exchange membrane; AC—bilayer membrane.

The electrochemical deposition of iron (III) and chromium (III) hydroxides was carried out in a 4-chamber cell (Figure 1). Electrodeposition was carried out in bilayer membranes with a cation-exchange layer thickness of 30 μm. To form a catalyst layer at the interface between the cation-exchange layer/anion-exchange layer, the membranes were kept in a 0.1 M NaOH solution to convert the anion-exchange layer of the membranes into the OH^−^ form. Electrodeposition was carried out under the following conditions: the concentration of salt and alkali solutions supplied to the cell was 0.1 M, the polarizing current density was 3 mA/cm^2^, the deposition time was 1 min for iron (III) hydroxide, and varied from one to ten minutes for chromium (III) hydroxides. In the latter case, a change in the deposition time made it possible to obtain catalyst particles of various degrees of dispersion.

The bilayer membranes obtained in this way were kept for 24 h in a 0.01 M sodium chloride solution to ripen the sediment, which further ensured the stability of the electrochemical characteristics.

#### 2.1.5. Deposition of Iron (III) Hydroxide Sol

Particles of iron (III) hydroxide sol with a size of 50–500 nm were applied to the surface of an anion-exchange membrane at a temperature of 100 °C by dipping the anion-exchange membrane into an iron (III) hydroxide sol for 10 s. After drying the membrane, a film of sulfonated polytetrafluoroethylene with a thickness of 30 μm was applied to its surface.

### 2.2. Electrochemical Measurements

Various methods can be used to study the process of water splitting and ion transport in bipolar membranes, many of which were first developed and applied to other electromembrane systems. Among these methods, the most important are voltammetry [74], chronopotentiometry [75], the method of the frequency spectrum of electrochemical impedance [76], and measurement of the number of ion transport through membranes.

In the present work, the measurements were carried out in a four-compartment electrochemical cell (Figure 1) with active membrane area 2.27 cm^2^. The Luggin–Haber capillaries connected to silver chloride electrodes were used to study the current-voltage characteristics and monitor the potential drop on the membrane during measurement of the transport numbers. The frequency spectrum of the impedance was measured with Pt/Pt electrodes made of platinum wire 0.1 mm in diameter. The large surface area of the platinized electrodes prevents their polarization when a direct current flows through the system. Measuring platinum electrodes and tips of the Luggin–Haber capillaries were located at a fixed distance from the membrane surface equal to 0.2 mm. Auxiliary membranes on both sides from the membrane under study prevent its contact with products of electrode reactions.

In most parts of the experiments, the membrane under investigation was in contact with hydrochloric acid solution from the side of the cation-exchange layer, and with sodium hydroxide solution from the side of the cation-exchange layer. This setup is labeled as “acid-alkali system”. This membrane setup is favoring the study of the water-splitting mechanism [76].

In other experiments, the membrane was in contact with sodium chloride solution from both sides. This setup is marked as “salt system”. This membrane setup is used for studying the salt ions transport across the bilayer membrane.

The solutions feed rate was 2 mL/min. The solutions and the cell were kept at a constant temperature 25 ± 0.1 °C.

#### 2.2.1. Voltammetry

A dynamic method of measuring the current-voltage characteristics was used. The test membranes were pre-conditioned for 30–40 min in an electrochemical cell (Figure 1), with the pump on and without current. After that, a linearly increasing current was applied to the cell with the help of a Potentiostat/Galvanostat/EIS Analyzer Parstat 4000, (AMETEK SI, Oak Ridge, TN, USA) and the current-voltage characteristic was recorded.

Current sweep rate was set at 2·10^−5^ A/s.

The effect of diffusion boundary layer thickness on ions transport across bilayer membranes were studied using a rotating membrane disk. The rotating membrane disk setup and methodology are explained in detail in a number of publications [77,78].

The method of voltammetry makes it possible to qualitatively study the process of ion transport through the BPM, in those cases when it is not necessary to know the overvoltage on each of the layers composing the BPM. The type of the current-voltage characteristic is determined by the structure of the membrane and by the nature of the processes occurring in it under polarization by an electric current.

**Figure 2:**
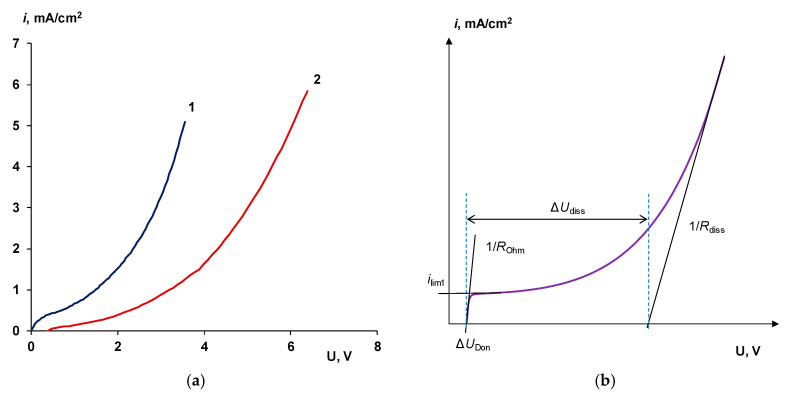
(**a**) Current-voltage characteristic of a bilayered membrane BM-a-30 measured in a 0.01 M NaCl solution (1) and in the 0.01 M acid-alkali system (2); (**b**) schematic current-voltage curve of a bipolar membrane in a salt solution.

Notably, the current-voltage characteristics of studied membranes differ in shape when measured in the salt system and in the acid-alkali system (Figure 2a). The current-voltage characteristic of a bipolar membrane in the acid-alkali system at small values of the polarizing current shows a significant deviation of the potential from zero. The reason for this is the emerging Donnan potential at the CEL/AEL interface. This potential shift allows for a qualitative assessment of the membrane’s catalytic activity towards the water splitting reaction. The closer this potential to the equilibrium, the higher the catalytic activity, and vice versa.

The current-voltage curve of the bilayer membrane with CEL/AEL interface can be conditionally divided into several sections (Figure 2b) [6], where each section is characterized by specific parameters. The first section of the current-voltage curve gives the ohmic resistance of the membrane system (*R*_Ohm_); the value of the first limiting current (*i*_lim1_) characterizes the current density carried by co-ions: the higher the membrane selectivity towards water splitting products, the lower *i*_lim1_. The length of the second section displays the potential drop at which an intense generation of H+ and OH- ions begins (Δ*U*_diss_), the so-called water splitting potential drop; the slope of the third section characterizes the water splitting resistance, (*R*_diss_), i.e., the ability to intensify water splitting: the smaller *R*_diss_, the higher the catalytic activity towards water splitting reaction [46,58].

The total potential drop on the membrane is the sum of several components: the Donnan potentials at the membrane-solution boundaries, the ohmic voltage drop across the monopolar layers, which is determined by the membrane structure and the type of charge carrier; potential drop at the bipolar boundary.

#### 2.2.2. Transport Numbers

Non-selective transfer of ions through the membrane is caused by two mechanisms: diffusion and migration. As a first approximation, it was assumed that the electromigration transport number *t_i_*^m^ is averaged over the thickness of the membrane and does not depend on the current density. The permeability coefficient *P_i_* was also assumed to be independent of the current density. Then, the effective transport number is the sum of two additives [6]:

(1) $$T_{i}=t_{i}^{m}+\frac{P_{j}\Delta c_{i}F}{iS},$$

Thus, the calculation of the average value of migration transport number of the hydrogen or hydroxyl ions through the membrane and the acid or alkali permeability coefficient decreases to determining the coefficients in the equation of the linear dependence of *T_i_* on the inverse current density *i*^−1^.

In this study, a modified Hittorf method was used. The essence of the method is to determine the total flux of sodium cations into the acid from alkali and total flux of chlorine anions into the alkali from acid.

At each investigated current density, the membrane was held for 60 min at a given current density to achieve a steady state. After that, a sample of acid and alkali solution was taken from the outlet of the cell. The sampling repeats every 20 min. After two consecutive measurements show a difference, not more than measurement error, the next current was applied to the cell.

The concentrations of ions in acid and alkali samples were measured using ion chromatography.

Effective transport numbers were calculated by the following equations:

(2) $$T_{{\mathrm{Na}}^{+}}=\frac{\left(c_{{\mathrm{Na}}^{+}}-c_{{\mathrm{Na}}^{+}}^{0}\right)WF}{iS},\;T_{{\mathrm{Cl}}^{-}}=\frac{\left(c_{{\mathrm{Cl}}^{-}}-c_{{\mathrm{Cl}}^{-}}^{0}\right)WF}{iS}$$

(3) $$T_{W}=1-\left(T_{{\mathrm{Cl}}^{-}}+T_{{\mathrm{Na}}^{+}}\right)$$

For simplicity, we consider the value of the transport numbers for hydrogen and hydroxyl ions to be the same. However, one must bear in mind that hydrogen ions can pass only through the cation-exchange layer and hydroxyl ions can pass only through the anion-exchange layer.

#### 2.2.3. Electrochemical Impedance Spectroscopy

Electrochemical impedance frequency spectra measurements were carried out in the frequency range of alternating current from 3 × 10^−3^ to 1 × 10^6^ Hz, distributed uniformly on a logarithmic scale. The amplitude of the AC signal was 200 mV.

The ohmic contribution of the total impedance of the cell itself is equal to 4 Ohm·cm^2^ for real and imaginary parts of impedance. The relative error magnitude of impedance measurements was less than 1%.

The method of the frequency spectrum of electrochemical allows obtaining the most complete information on the processes of ion transport through bilayer membranes under conditions when a direct current flows through the membrane.

**Figure 3:**
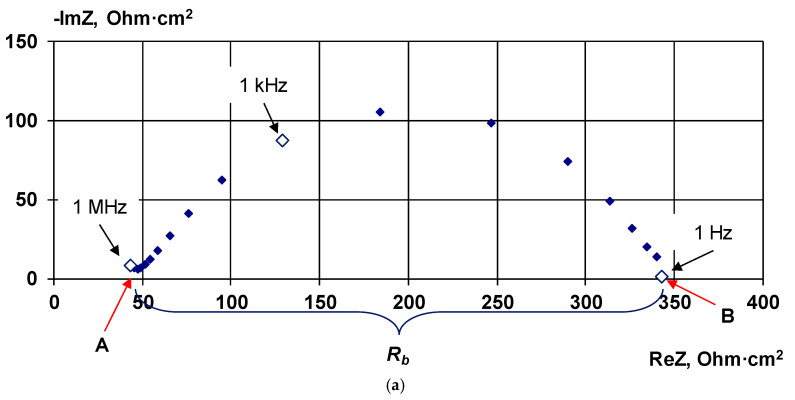

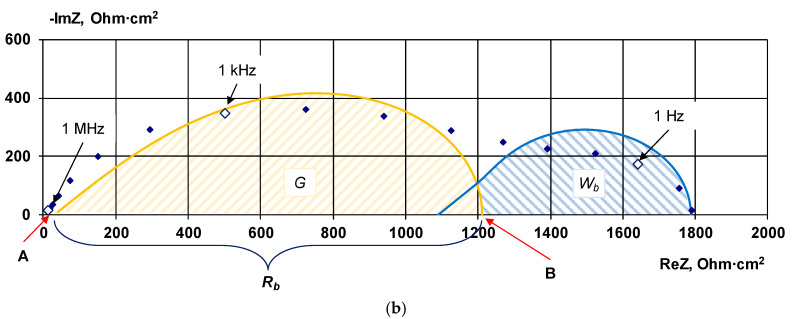
Electrochemical impedance spectrum of a bipolar membrane under DC polarization with superimposed AC amplitude. Examples based on the real spectra obtained for the BM-ac-2K membrane measured in 0.01 M “acid-alkali system” (**a**) and measured in 0.5 M NaCl solution (**b**).

In dilute solutions or at high current density, the impedance spectrum of the bilayer membrane is reduced to the impedance spectrum of the bipolar membrane. An example of the electrochemical impedance spectrum of the bilayer membrane in the so-called “generation mode” is shown in Figure 3a.

The limit of the frequency spectrum on the axis of active resistances at *ω* → ∞ is the ohmic part of total resisitance *R*_∞_ (point A). It includes the resistances of the monopolar layers, solutions between the membrane surface and the measuring electrode, electrodes themselves and all intermediate resistances associated with the wires and their connections. The second limit is the point B in which *ω* → 0 and the active resistance (*R*_0_) includes, in addition to the above, also the resistance caused by the slow chemical reaction at the bipolar boundary. The emergence of this resistance causes the appearance of the potential “excess” (overvoltage) of the bipolar region and leads to the deviation of the current-voltage characteristic from linearity. The resistance of the bipolar region (*R_b_*) can be found as:

(4) $$R_{b}=R_{0}-R_{\infty }$$

Integrating the resulting dependence of *R*_b_ from the current density, one can calculate the overvoltage of the bipolar region (*η_b_*):

(5) $$\eta_{b}=\int_{0}^{i}R_{b}di$$

In the case of a low current density, a thin cation-exchange layer, or a high concentration of external solutions, the electrochemical impedance spectrum of the bilayer membrane is a combination of two distorted semicircles (Figure 3b). The analysis of the impedance of ion-exchange membranes in electromembrane systems complicated by the course of chemical reactions was carried out in [79,80]. The first semicircle lying in the mid-frequency region (100 kHz–10 Hz), as in the case of bipolar membranes, is the Gerischer impedance (element G). The second semicircle, which appears at frequencies below 10 Hz, refers to the diffusion of salt ions through the bilayer membrane. This region in the spectrum is described by the finite Warburg impedance (element W_b_).

**Scheme 1:**
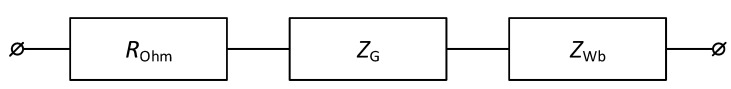
The equivalent circuit of the electrochemical impedance of an asymmetric bipolar membrane. *R*_Ohm_ is the ohmic component of the resistance of an electromembrane system, *Z_G_* is the Gerischer impedance, *Z*_Wb_ is the finite Warburg impedance

It was shown in [29] that the impedance spectrum of an asymmetric bipolar membrane (a particular case of a bilayer membrane) can be qualitatively described in terms of equivalent circuits as a series connected ohmic resistance, Gerischer and finite Warburg’s elements (Scheme 1).

The overall system impedance can be represented as:

(6) $$Z_{T}=Z_{\mathrm{Ohm}}+Z_{G}+Z_{W_{b}},$$

where:

(7) $$Z_{\mathrm{Ohm}}=R_{CEL}+R_{AEL},$$

(8) $$Z_{G}=\frac{R_{b}}{\sqrt{2}}\left(\sqrt{\frac{1}{\sqrt{a}}+\frac{1}{a}}-j\sqrt{\frac{1}{\sqrt{a}}-\frac{1}{a}}\right),$$

(9) $$Z_{W_{b}}=\frac{W_{s}}{\sqrt{\omega }}\left(1-j\right)\tan h\left(W_{c}\sqrt{j\omega }\right),$$

In the Gerischer impedance (Equation (8)): $a={\left(\frac{\omega }{\chi }\right)}^{2}+1$ and *χ* is the non-equilibrium effective rate constant of the water splitting reaction.

In the finite Warburg impedance (Equation (9)): *W_s_* is the Warburg’s coefficient; $W_{c}=\frac{d_{c}}{\sqrt{D_{c}}}$ is the characteristic length (*d_c_* is the thickness of the CEL, *D_c_* is the diffusion coefficient of co-ion (chloride ion) in the CEL).

To find the magnitude of the overvoltage of the bipolar region in the case of spectra complicated by the diffusion of salt ions, we used the fitting of the parameters of the equivalent circuit to the experimental data. The found parameters of the elements of the equivalent circuit were used to simulate the individual impedance components (Equations (8) and (9)). After finding the bipolar resistance (*R*_b_), the bipolar overvoltage can be found using Equation (5).

## 3. Results

### 3.1. Cation-Exchange Layer Properties Thickness and Chemical Nature

As shown in a number of works [24,28,81], for the formation of a bipolar boundary, it is sufficient to apply a cation-exchange layer of a very small thickness (10–50 μm) to the substrate membrane.

**Figure 4:**
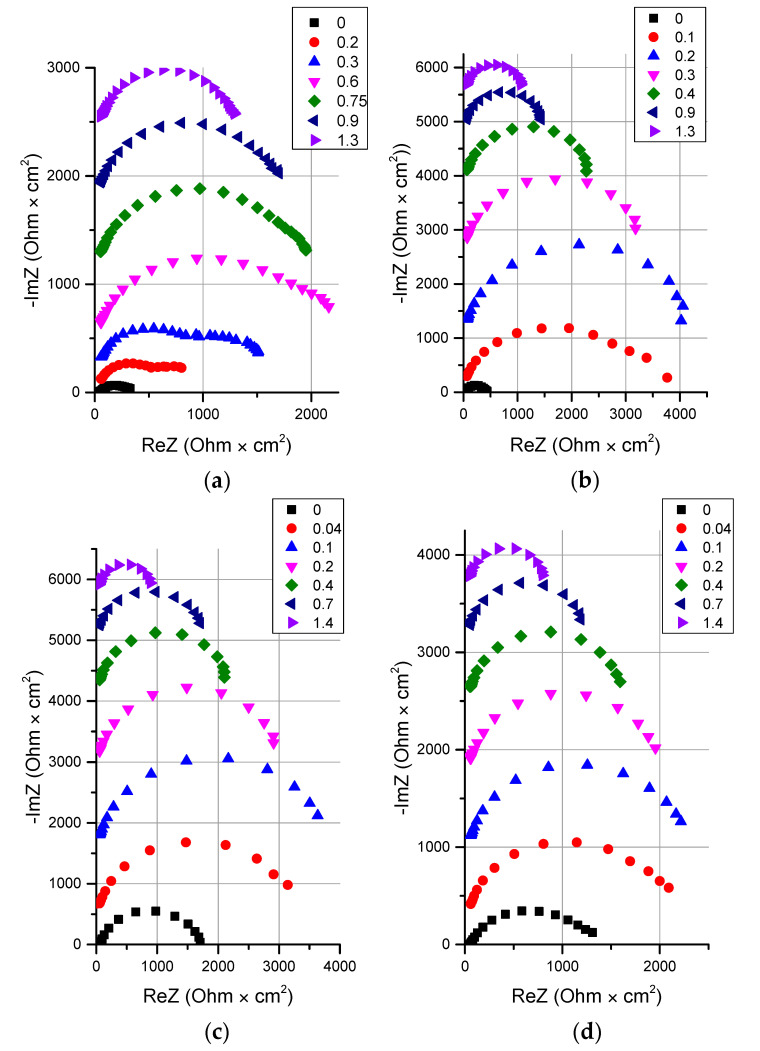
Electrochemical impedance spectra of bilayer membranes BM-a measured in the “acid-alkali system” (concentrations of the solutions are 0.01 M). The CEL thickness is (microns): (**a**)—10, (**b**)—30, (**c**)—50, (**d**)—70. Legends show the DC current density values in mA/cm^2^. The individual plots are shifted vertically for better clarity of presentation.

The effect of the cation-exchange film applied to the surface of the anion exchange membrane is primarily manifested in the electrochemical impedance spectra (Figure 4).

The impedance spectrum of a bilayer membrane in dilute solutions takes the form characteristic of a spectrum of a bipolar membrane [82,83]. For all the studied bilayer membranes of BM-a series, a general pattern of their change with increasing current density is observed. The minimum impedance value at zero current density is explained by the presence of salt ions at the bipolar boundary. With an increase in the current density, the impedance spectrum of the membrane expands, which is associated with the removal of salt ions (charge carriers) from the bipolar region and an increase in its resistance up to a certain maximum. With a further increase in the current density, the spectrum gradually narrows, since the water splitting reaction proceeding at the bipolar boundary leads to the appearance of new charge carriers that are hydrogen and hydroxyl ions.

In the absence of a catalyst for the water splitting reaction, the resistance of the bipolar region can reach several hundred, and at low current densities, several thousand Ohm·cm^2^. In this case, the contribution of the resistance of the monopolar regions (values of the impedance at high frequencies) is insignificant (about 50 Ohm·cm^2^), even in dilute solutions.

**Figure 5:**
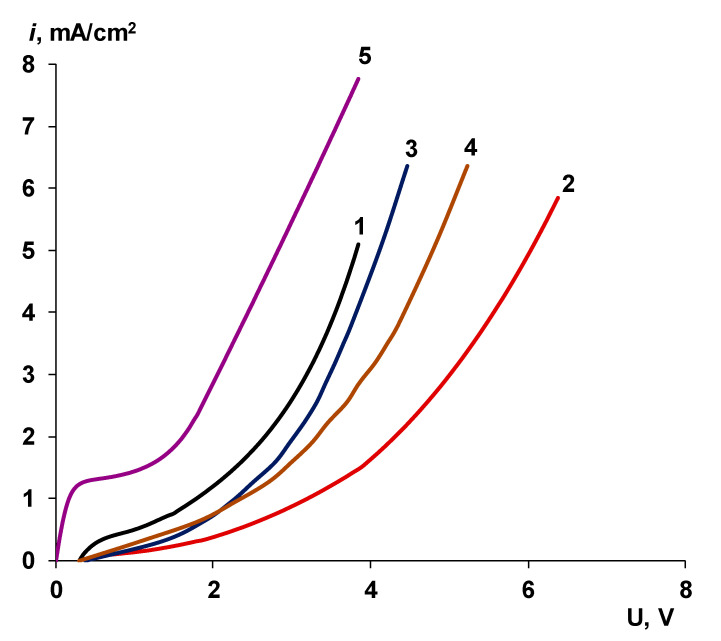
General current-voltage characteristics of the BM-a membranes in the “acid-alkali system”. The CEL thickness is (microns): 1–10, 2–30, 3–50, 4–70; 5–unmodified Ralex AMH. Acid and alkali concentrations are 0.01 M.

From the general current-voltage characteristics, it can be seen that despite the small thickness of the modifying film (Figure 5, curves 1, 2), the value of the limiting current density decreases by more than an order of magnitude compared with the anion-exchange membrane-substrate (Figure 5, curve 5).

The increase of the current density higher than the limiting value leads to the formation of a space charge region at the CEL/AEL interface and the beginning of the water-splitting reaction. The appearance of new charge carriers (hydrogen and hydroxyl ions) at the bipolar border leads to a deviation of the shape of the current-voltage characteristic from the linearity of the current limiting plateau. The following rise of current appears exclusively due to water splitting reaction and can be addressed to the flux of H^+^ and OH^−^ ions [84]. For a bipolar membrane close to “ideal”, the potential at which the water splitting begins can be close to the thermodynamic value of 0.83 V.

**Figure 6:**
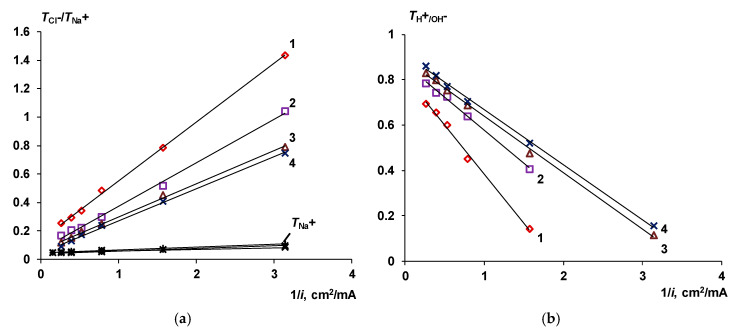
Dependence of transport numbers of chloride and sodium ions (**a**) and hydrogen and hydroxyl ions (**b**) for bilayered membranes BM-a on inverse current density measured in the “acid-alkali system”. Thickness of the CEL, microns: 1–10, 2–30, 3–50, 4–70. Acid and alkali concentrations are 0.01 M.

Direct measurement of the transport numbers of salt ions and hydrogen and hydroxyl ions through the studied membranes depending on the thickness of the cation-exchange layer shows that with an increase in the thickness of the cation-exchange layer, the transport number of chloride-ion decreases (Figure 6a). Moreover, when the cation-exchange layer’s thickness is 50 μm or higher, the transport numbers’ differences become insignificant. Their values are comparable to the values obtained for the MB-2 bipolar membrane.

The transport numbers of the sodium ions in all cases weakly depend both on the applied current density and on the properties of the cation-exchange layer. In all cases, its value does not exceed 0.1, since the transport numbers of the sodium ion are completely determined by the properties of the anion-exchange layer, and they, in this case, remain unchanged.

The values of the limiting current density, electromigration transport numbers, and the water splitting potential obtained in the acid-alkali system for BM-a series membranes are given in Table 2.

#### Chemical Nature of the CEL

Let us estimate how the chemical nature of the cation-exchange layer affects the transport of salt ions and the rate of water splitting. The main issue to be solved is the dependence of the limiting current (and, accordingly, selectivity) on the chemical nature of the cation-exchange layer. In this regard, studies were carried out in the “salt system” with a 0.02 M NaCl solution.

**Figure 7:**
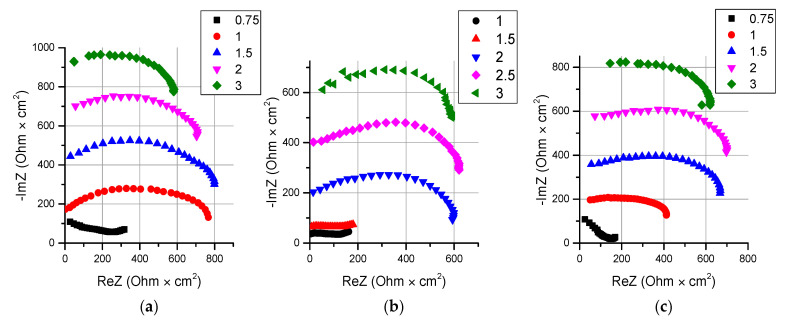
Electrochemical impedance spectra of bilayer membranes with different chemical nature of the CEL measured in 0.02 M NaCl. (**a**)—sulfonated polytetrafluoroethylene, (**b**)—sulfonated polysterene, (**c**)—sulfonated poly(ether ether)ketone. Legends show the DC current density values in mA/cm^2^. The individual plots are shifted vertically for better clarity of presentation.

The electrochemical impedance spectra of bilayer membranes obtained by applying a thin (30 μm) cation-exchange layer of various chemical nature to the Ralex AMH substrate membrane are shown in Figure 7.

**Figure 8:**
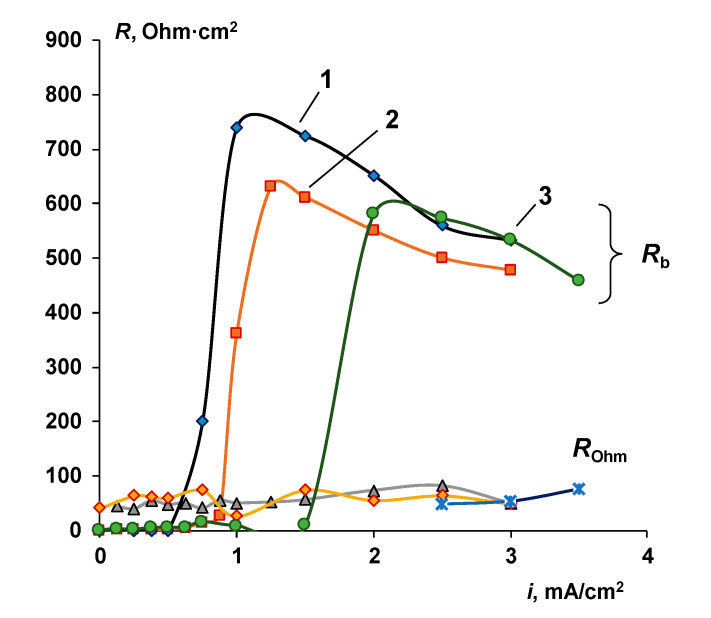
Dependence of bipolar region and ohmic resistances on current density for bilayer membranes with different chemical nature of the CEL: 1—sulfonated polytetrafluoroethylene, 2—sulfonated poly(ether ether)ketone, 3—sulfonated polysterene.

Due to the high flux of salt ions in a saline solution, the electrochemical impedance spectra of bilayer membranes are a combination of two impedances (Gerischer impedance and finite Warburg impedance), which is why the shape of the spectra is greatly distorted. The obtained spectra were analyzed using the equivalent circuit fitting method. The obtained values of the resistance of the bipolar region and monopolar layers of the studied membranes are shown in Figure 8. It can be seen from the figure that a sharp increase in the resistance of the bipolar region, which corresponds to the moment when mobile ions (salt ions) are removed from it and the space charge region is formed, increases in the following row: sulfonated polytetrafluoroethylene < sulfonated poly(ether ether)ketone < sulfonated polysterene. The formation of the space charge region at higher currents indicates a high permeability of the cation-exchange film for salt ions. In the case of an SPS film, this may be due to the instability of the cation-exchange layer made of a water-soluble polyelectrolyte.

The ohmic part of the resistance of all three membranes is close to each other and is approximately equal to 50 Ohm·cm^2^ as in the case of the acid-alkali system.

The found values of the effective water splitting reaction rate constants are shown in Table 3.

The found values of the effective water splitting reaction rate constants in over-limiting current modes confirm that membrane with a cation-exchange layer made of sulfonated polytetrafluoroethylene is the most effective in the water splitting reaction, which is associated with the highest selectivity of this sample in comparison with membranes with other cation-exchange coatings.

### 3.2. Catalyst Effect on the Water Splitting Kinetics

**Figure 9:**
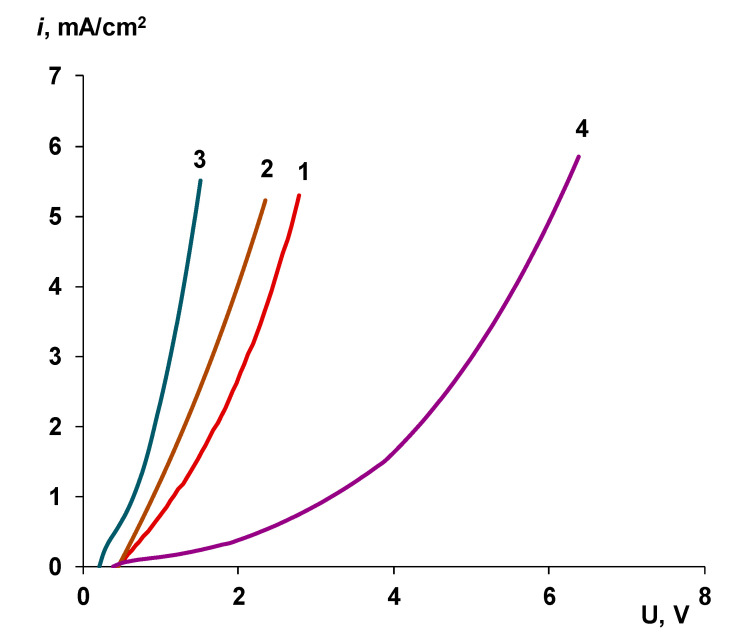
General current-voltage characteristics of the BM-ac membranes with KF-1 catalyst in “acid-alkali system”. Amount of the catalyst (mg/cm^2^): 1—0.2, 2—2, 3—6; 4—the BM-a-30 membrane. Acid and alkali concentrations are 0.01 M.

**Figure 10:**
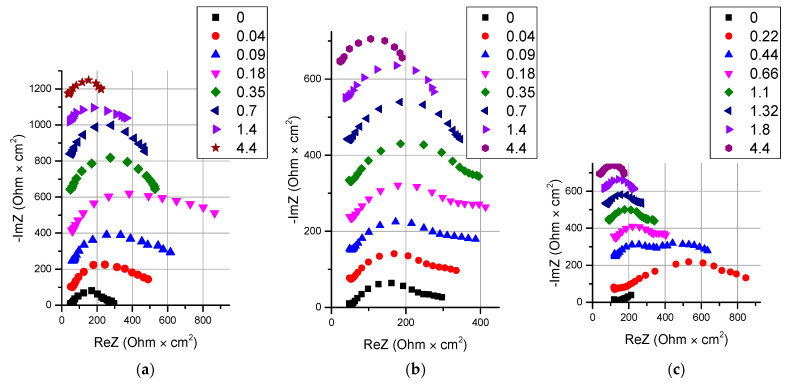
Electrochemical impedance spectra of bilayer membranes BM-ac with KF-1 catalyst measured in the “acid-alkali system” (concentrations of the solutions are 0.01 M). The amount of catalyst is (mg/cm^2^): (**a**)—0.2, (**b**)—2, (**c**)—6. Legends show the DC current density values in mA/cm^2^. The individual plots are shifted vertically for better clarity of presentation.

Figure 9 shows the current-voltage characteristics, and Figure 10 shows the frequency spectra of the electrochemical impedance of bilayer membranes with different contents of a phosphoric acid catalyst (KF-1). It follows from the analysis of the current-voltage curves that even small amounts (0.2 mg/cm^2^) of the catalyst are sufficient for a sharp decrease in the water splitting potential drop. At a high catalyst content, a plateau of the limiting current appears on the general current-voltage characteristics (Figure 9, curve 3). For the same membrane, the distortion of the spectrum appears at low frequencies due to the electrodiffusion of salt ions (Figure 10c).

The shift of the current-voltage characteristic towards a lower water splitting potential drop with an increase in the amount of catalyst is apparently associated with an increase in the area of the bipolar contact between the particles of the catalyst and the anion exchanger.

Measurement of the electrochemical impedance of the BM-ac series membranes shows that, upon the introduction of a catalyst, the impedance of the bipolar region decreases by almost an order of magnitude in comparison with the BM-a series membranes. In addition, for these membranes, there is no characteristic dependence of the spectrum width on the magnitude of the direct current superimposed on the membrane. With an increase in the current density, there is no sharp expansion of the spectrum with its subsequent gradual narrowing. In this case, after expansion, the spectrum retains its shape in a wide range of current densities, which indicates a high catalytic activity of the ion-polymer particles and, in particular, that the water splitting reaction occurs when even a small direct current is applied.

In the region of low currents, a deviation of the spectrum shape from the Gerischer impedance is observed. Moreover, an increase in the amount of catalyst to 6 mg/cm^2^ leads to the formation of the Warburg impedance at low currents.

**Figure 11:**
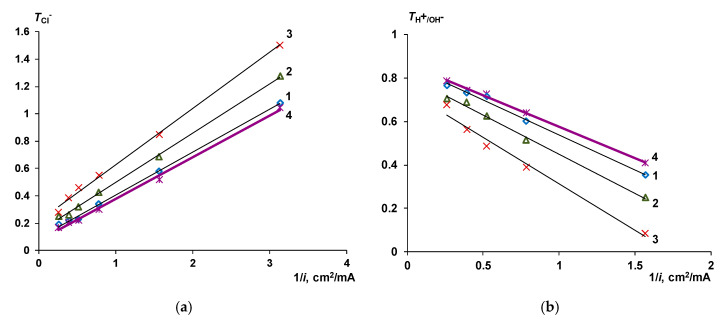
Dependence of transport numbers of chloride (**a**) and hydrogen/hydroxyl (**b**) ions for bilayer membranes BM-ac with KF-1 catalyst on inverse current density measured in the “acid-alkali system”. The amount of catalyst is (mg/cm^2^): 1—0.2, 2—2, 3—6; 4—the BM-a-30 membrane. Acid and alkali concentrations are 0.01 M.

The study of the transport numbers of chloride and sodium ions showed that the chloride ion’s transport numbers are higher in the entire range of current densities. The transport numbers of the water splitting products are lower than for the membrane without a catalyst (Figure 11).

The values of the limiting current density, electromigration transport numbers, and the water splitting potential drop obtained in the acid-alkali system for BM-ac membranes with KF-1 catalyst are given in Table 4.

#### 3.2.1. Chemical Nature of the Catalyst

Despite the high activity in the water splitting reaction, the use of the ion-polymer catalyst KF-1 is limited due to a decrease in the transport numbers of hydrogen and hydroxyl ions. The reasons for this decline will be discussed below in Section 4.

**Figure 12:**
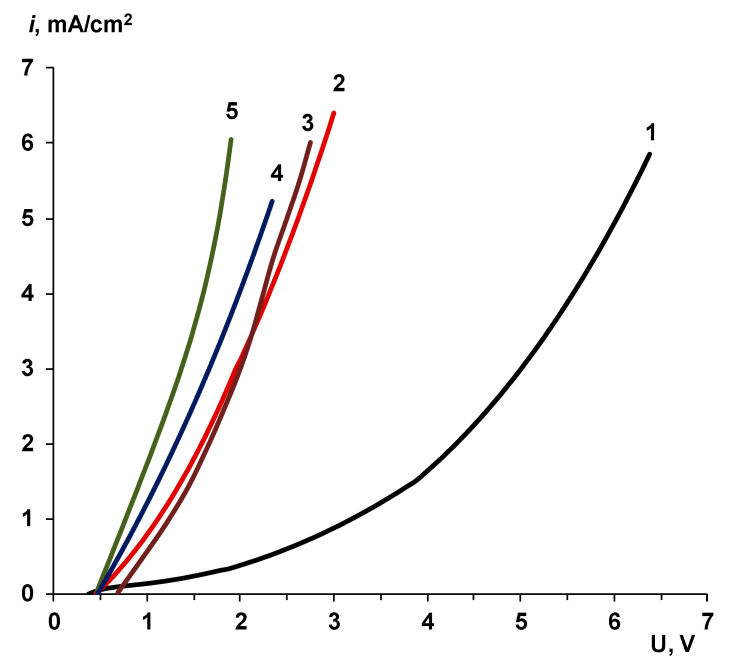
General current-voltage characteristics of the BM-ac membranes with various catalysts measured in the “acid-alkali system”. Membranes: 1—BM-a-30, 2—BM-ac-2dFe, 3—BM-ac-solFe, 4—BM-ac-2K, 5—BM-ac-2dCr. Acid and alkali concentrations are 0.01 M.

**Figure 13:**
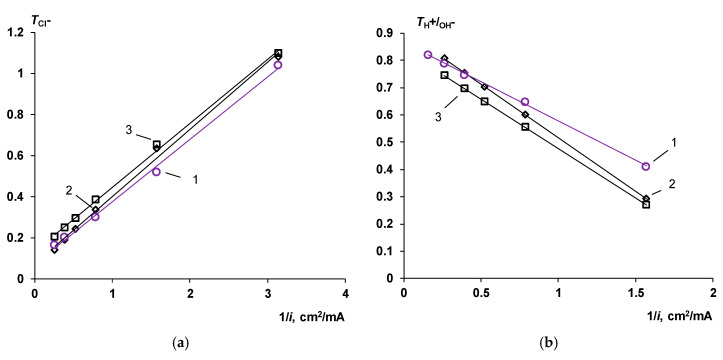
Dependence of transport numbers of chloride (**a**) and hydrogen/hydroxyl (**b**) ions for bilayer membranes BM-ac with hydroxide catalyst on inverse current density measured in the “acid-alkali system”. Membranes: 1—BM-a-30, 2—BM-ac-2dCr, 3—BM-ac-2dFe. Acid and alkali concentrations are 0.01 M.

The introduction of iron (III) and chromium (III) hydroxides into the cation-exchange layer of a bilayer membrane can significantly reduce the water splitting potential drop (Figure 12), practically without changing the transport numbers of chloride ions (Figure 13).

The weighted average particle size of the catalysts is 10–20 micrometers (Supplementary Figure S1). The operating voltages across the membranes with catalysts are 1.76, 2.25, and 2.56 V at a current density of 5 mA/cm^2^ in the series Cr(OH)_3_, KF-1, Fe(OH)_3_. Among the three indicated catalysts, chromium (III) hydroxide shows the best characteristics, which coincides with the extended row of water splitting activity proposed in [69].

An increase in the iron (III) hydroxide catalyst dispersion due to the use of the sol particles (particle size 50–500 nm) does not lead to significant changes in the current-voltage characteristic (Figure 12, curve 3).

#### 3.2.2. Influence of the Particle Size of Chromium (III) Hydroxide on the Rate of the Water Splitting Reaction

As noted in the previous section, the membrane containing chromium (III) hydroxide showed the best electrochemical characteristics. At the same time, there is a way to improve the electrochemical characteristics to reduce the water splitting potential drop and increase the speed of the reaction by increasing the degree of dispersion of the catalyst particles.

**Figure 14:**
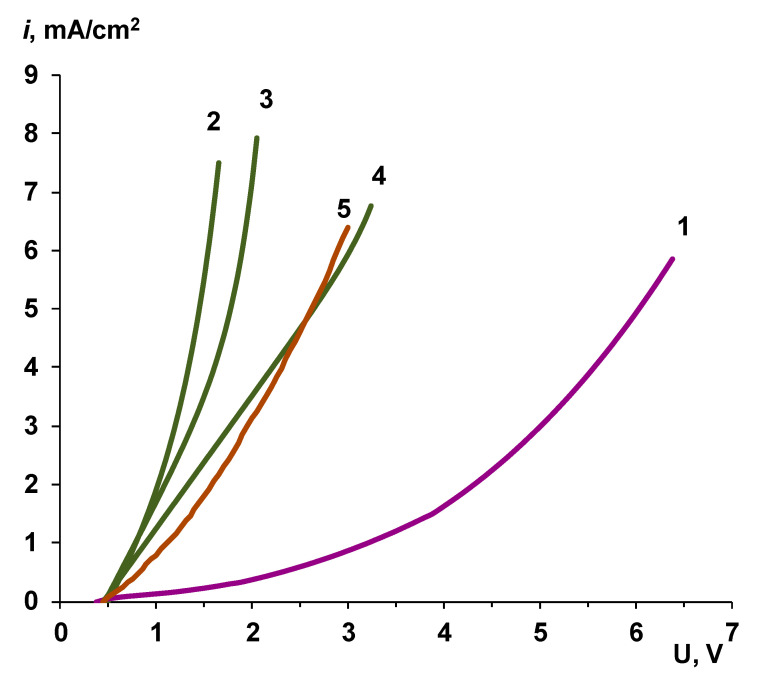
General current-voltage characteristics of the BM-ac membranes with electrodeposited hydroxides measured in the “acid-alkali system”. Membranes: 1—BM-a-30, 2—BM-ac-1Cr, 3—BM-ac-2Cr, 4—BM-ac-10Cr, 5—BM-ac-1Fe. Acid and alkali concentrations are 0.01 M.

Figure 14 shows that with decreasing deposition time, i.e., an increase in the degree of particle dispersion, a greater decrease in the working voltage on the membrane occurs. This can be explained by the fact that, with an increase in the electrodeposition time over 2 min, the catalyst does not form separate particles, but a continuous layer at the bipolar boundary. The electrical conductivity of such a layer is lower in comparison with the electrical conductivity of the membrane, and the generating surface is smaller. The combined effect of these factors leads to an increase in the membrane resistance and the water splitting potential drop.

The use of catalysts obtained by electrochemical deposition of particles on the bipolar boundary makes it possible to improve the characteristics of membranes with chromium (III) hydroxide (at a current density of 5 mA/cm^2^, the voltage drop across the membrane decreases from 1.76 to 1.45 V compared with membrane with 2 mg/cm^2^ microdispersion of chromium (III) hydroxide). For the membrane with electrodeposited iron (III) hydroxide, no significant changes in the operating voltage between the microdispersed form and the electrodeposited form of the catalyst were found.

**Figure 15:**
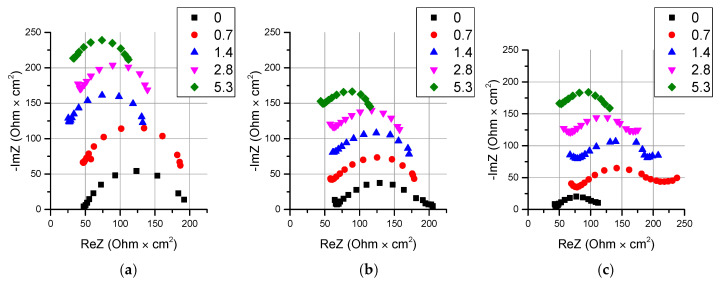
Electrochemical impedance spectra of bilayer membranes BM-ac with electrodeposited Cr(OH)_3_ catalyst measured in the “acid-alkali system” (concentrations of the solutions are 0.01 M). The electrodeposition time is (min): (**a**)—1, (**b**)—2, (**c**)—10. Legends show the DC current density values in mA/cm^2^. The individual plots are shifted vertically for better clarity of presentation.

Figure 15 shows the results of a study of the electrochemical impedance of membrane samples with electrodeposited chromium (III) hydroxide. The presented spectra show that the introduction of chromium (III) hydroxide leads to comparable changes in the impedance of the bipolar region, regardless of the deposition time. The impedance of the bipolar boundary in all cases is approximately 70–150 Ohm⋅cm^2^. In the case when the electrodeposition was carried out for more than 2 min, a decrease in the impedance of the bipolar region in the absence of polarization (0 mA/cm^2^) can be observed. The reason for this can be the loosening of the surface cation-exchange film by a layer of chromium (III) hydroxide and an increase in the diffusion transfer of salt ions through the membrane.

The formation of a continuous layer of chromium (III) hydroxide with an increase in electrodeposition time is confirmed by changes of the ohmic resistance of the membrane. Its values increase with increase of the electrodeposition time, from 50 Ohm⋅cm^2^ (1 min electrodeposition) to 65 Ohm⋅cm^2^ and 78 Ohm⋅cm^2^ (2 and 10 min) under 0 mA/cm^2^ DC polarization.

Distortions in the spectra of membranes deposited for 10 min (Figure 15c) can be attributed to the diffusion of salt ions across the membrane, as in the case of a membrane containing an ion-polymer catalyst. However, due to the high activity of chromium (III) hydroxide particles in the water splitting reaction, the transport of salt ions is suppressed even at low current densities.

### 3.3. Electrochemical Properties in Solutions with High Concentrations

Since, starting with a cation-exchange layer thickness of 50 μm, bilayer membranes acquire selective properties close to ordinary bipolar membranes; membranes with a cation-exchange layer 30 μm thick were chosen to study the transport of ions in concentrated solutions.

**Figure 16:**
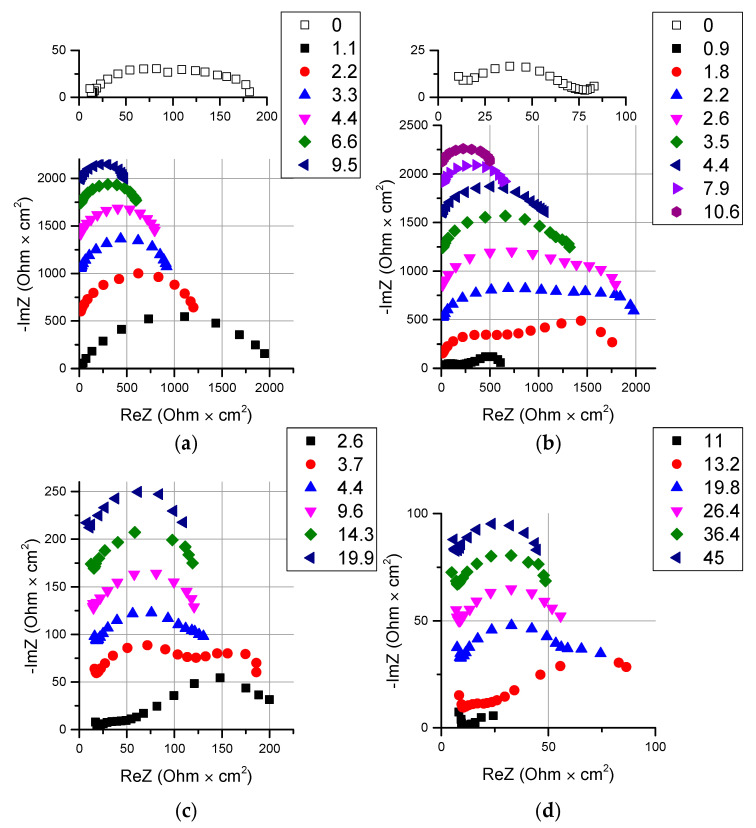
Electrochemical impedance spectra of bilayer membranes BM-a-30 (**a**,**b**) and BM-ac-2K (**c**,**d**) in the “acid-alkali system”. Concentrations of the solutions are: (**a**,**c**)—0.1 M, (**b**,**d**)—0.5 M. Legends show the DC current density values in mA/cm^2^. The individual plots are shifted vertically for better clarity of presentation.

The electrochemical impedance spectra of a bilayer membrane with a cation-exchange layer thickness of 30 μm in a 0.1 M acid-alkali system (Figure 16a) do not fundamentally differ from the spectra obtained in 0.01 M solutions (Figure 4b). In the spectra obtained in 0.5 M solutions (Figure 16b), a distortion in the low frequency region of the hodograph semicircle appears, which indicates salt ion electrodiffusion through the CEL.

It is interesting to note that with an increase in the concentration of external solutions, the electromigration transport numbers through the membrane practically do not change, and the voltage of the beginning of water splitting and the value of the limiting current increase (Table 5).

The transport number of water splitting products decreases with an increase in the concentration of solutions from 0.01 M to 0.5 M and from 0.86 to 0.82 for the BM-a-30 membrane. Considering that the main ions that lower the membrane selectivity are chloride anions, it can be concluded that, at sufficiently high current densities, the concentration of the external solution does not matter, and the main transport process is water splitting. At the same time, the current density, at which the water splitting plays a significant role in the overall mass transfer, increases with the increasing concentration of the solution. At low current densities, water splitting does not occur at the bipolar boundary, and the bilayer membrane behaves as a monopolar one, at the same time capable of selective transport of monovalent ions. In solutions with a concentration of 0.5 M at a current density of 10 mA/cm^2^, the transport number of hydrogen and hydroxyl ions is 0.4, while in more dilute solutions, at the same current density, it is 0.7–0.9.

When the water splitting reaction in the form of KF-1 microdispersed powder is introduced into the cation-exchange layer, a trade-off is observed between the water splitting voltage and limiting current (selectivity). Thus, the water splitting potential drop on the BM-ac-2K membrane increases from 1.2 to 2.3 V with an increase in concentration from 0.01 to 0.5 M. For the BM-a-30 membrane in the same solutions, the voltage increases from 3.7 to 10 V. However, at 0.5 M in the acid-alkali system, the limiting current on the membrane with a catalyst is five times higher than on the membrane without a catalyst (3.1 mA/cm^2^ for BM-a-30 and 15 mA/cm^2^ for BM-ac-2K). At certain systems (when the transport of salt ions and simultaneous water splitting is required), this fact leads to a preferable use of membrane with catalyst.

Thus, the membranes under study, when operating in moderately concentrated solutions, are capable of simultaneous desalination of the solution and the generation of water splitting products.

### 3.4. Thin Anion-Exchange Layer

**Figure 17:**
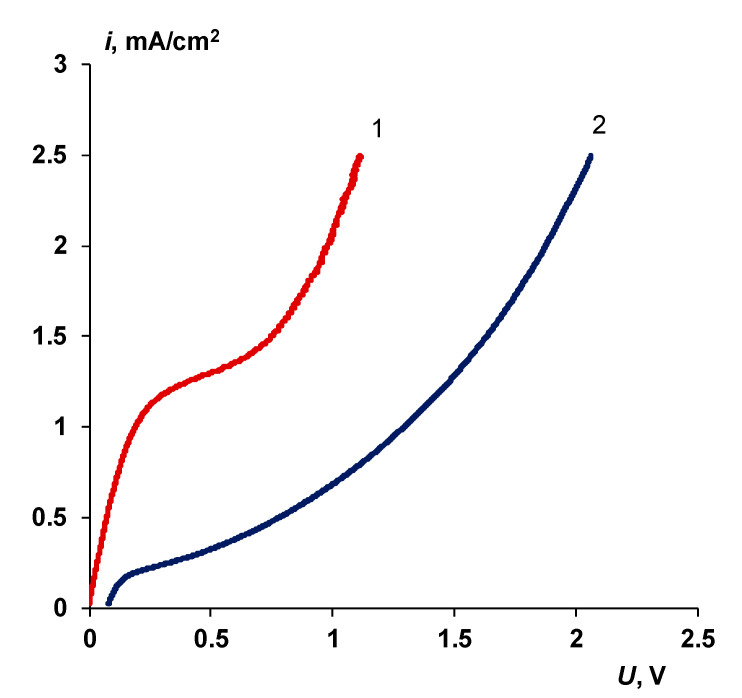
General current-voltage characteristics of the membranes measured in the “acid-alkali system”. Membranes: 1—Membrane-substrate Ralex CM, 2—BM-c-A80. Acid and alkali concentrations are 0.01 M.

**Figure 18:**
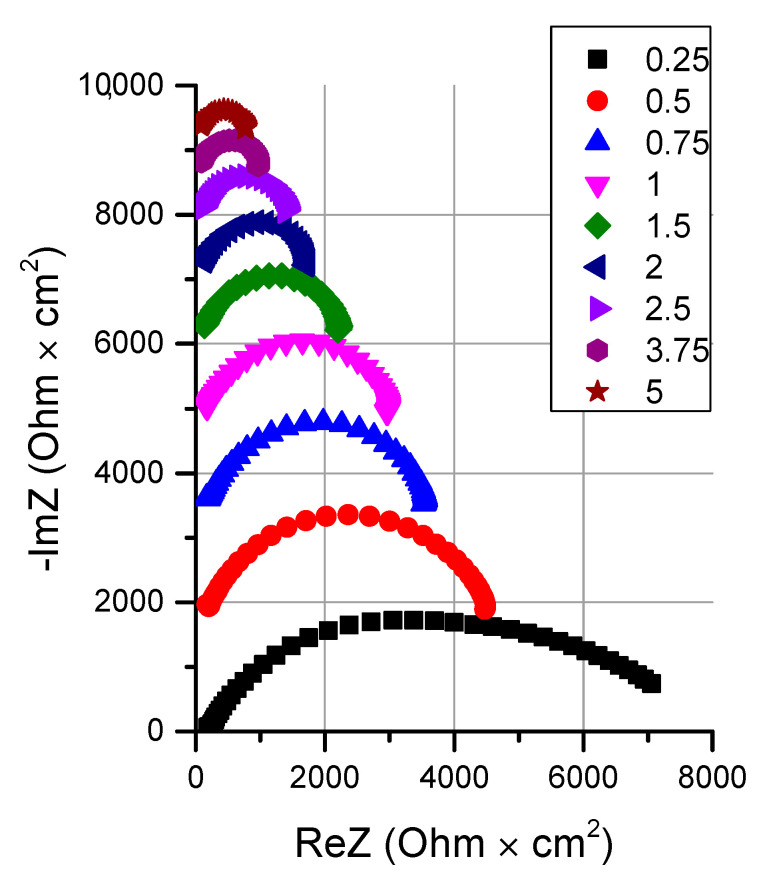
Electrochemical impedance spectra of bilayer membranes BM-c-A80 measured in the “acid-alkali system” (concentrations of the solutions are 0.01 M). Legends show the DC current density values in mA/cm^2^. The individual plots are shifted vertically for better clarity of presentation.

When an anion-exchange film is applied to the surface of a cation-exchange membrane, the same effects are observed as in the case of the BM-a series membranes (Figure 17). The value of the limiting current decreases sharply. At the CEL/AEL interface, as in the case of BM-a bilayer membranes, the water splitting reaction proceeds, as evidenced by the nature of the change in the electrochemical impedance spectra of the obtained membrane (Figure 18).

From the results obtained, it can be concluded that the regularities of ion transfer obtained for membranes with a thin cation-exchange layer will also be fulfilled for membranes with a thin anion-exchange layer and a cation-exchange substrate membrane.

### 3.5. Comparison with Commercial Bipolar Membranes

**Figure 19:**
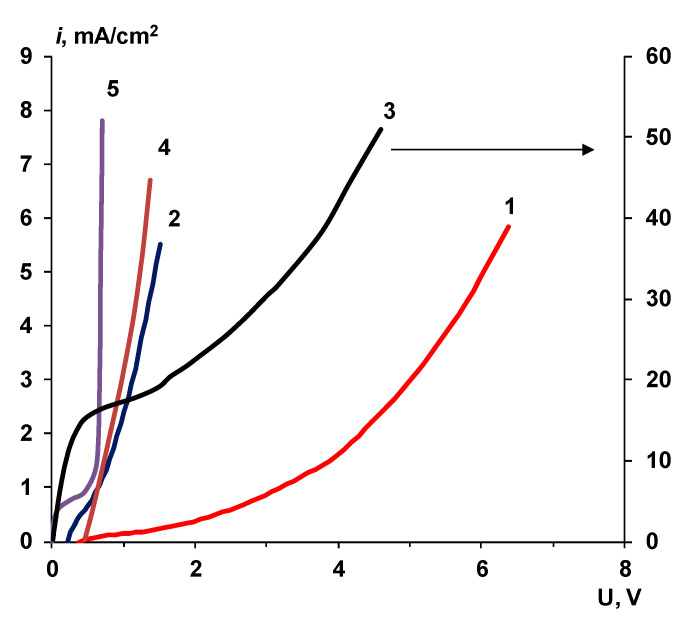
General current-voltage characteristics of bilayer and bipolar membranes. 1—BM-a-30 (0.01 M acid-alkali); 2—BM-ac-2K (0.01 M acid-alkali); 3—BM-ac-2K (0.5 M NaCl), 4—bipolar membrane MB-3 (0.01 M acid-alkali); 5—bipolar membrane BP-1 (2 M NaCl (adapted from [28])).

A direct comparison of membranes with high activity in the water-splitting reaction (BM-ac, MB-3, BP-1) in this paper is not possible, since the conditions in which curves 3 and 4 were obtained from the conditions for curve 5 are significantly different (Figure 19).

The introduction of the catalyst (Figure 19, curve 2) significantly reduces the operating voltage on the bilayer membrane. The current-voltage characteristic of the BM-ac membrane is close to the current-voltage characteristic of the MB-3 membrane. Both membranes contain phosphoric ion-exchange groups in the cation-exchange layer, which explains the similarity of their features. In the case of an MB-3 membrane (Figure 19, curve 4), the ohmic region and the plateau of the limiting current are absent, due to high activity towards water splitting, especially in dilute solutions. In case of solutions with a high concentration (1 M and higher), the current limiting plateau can manifest even for the very selective BP-1 membrane (Figure 19, curve 5).

As already mentioned, the presence of an ohmic section on the current-voltage characteristic of a bipolar membrane indicates the transfer of salt ions. In the case of the standard application of bipolar membranes (production of concentrated acid and base), the transfer of salt ions is an undesirable process, because it reduces the current efficiency of the electrodialysis process and leads to contamination of the product. We can see that when the concentration of the external solution is 0.5 M, the ohmic region for the BM-ac-2K membrane is significantly higher than for the common bipolar membranes like MB-3 and BP-1 (Figure 19, curve 3). This fact makes this membrane not very attractive for the direct production of alkali and acid. At the same time, for operations in which simultaneous desalination and pH correction are required, such membranes may be preferable to bipolar membranes.

In the diluted solutions, these membranes show characteristics comparable to the conventional bipolar membranes [30]. They can find application in the processes where acid and alkali concentration are not the main criteria. The production of deionized water is one example.

## 4. Discussion

In this section, we will focus on the two most interesting phenomena, which are derived from the experiments. The first one is the nature of the limiting current density found for bilayer membranes. The second one is the kinetics of the water-splitting reaction in the system with bilayer membranes.

### 4.1. The Nature of the Limiting Current on Bilayer Membrane

The found values of the limiting currents (*i*_lim_) are significantly lower than the values found for the substrate membrane. Moreover, with an increase in the thickness of the cation-exchange layer from 10 to 30 μm, this discrepancy increases.

**Figure 20:**
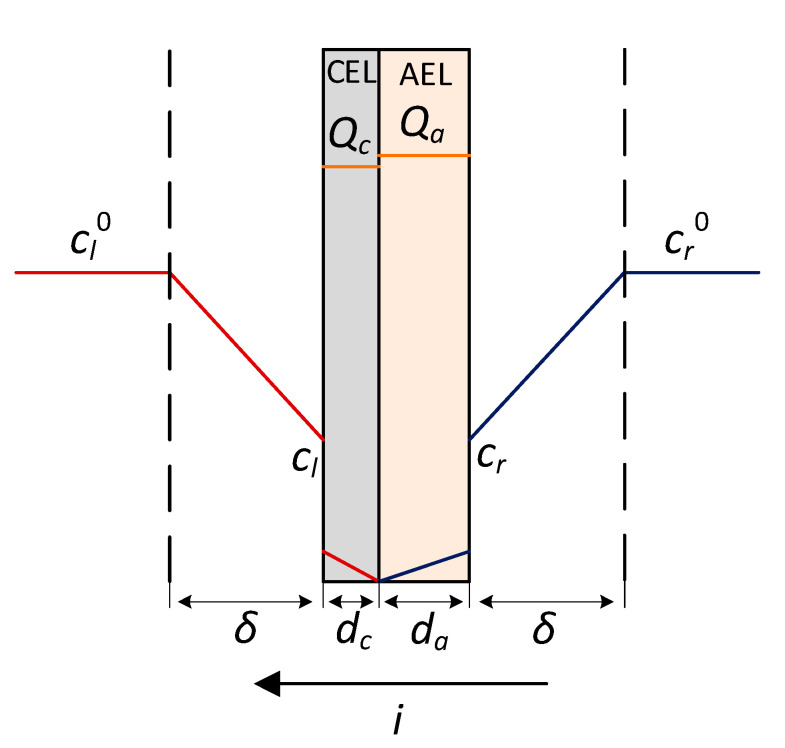
Schematically drawing showing concentration profiles under direct current polarization.

Let us consider the studied BM-a series bilayer membranes as a special case of bipolar ones. The concentration profiles for salt ions in the underlimiting current mode are shown in Figure 20.

The membrane is in contact with two solutions with equal concentrations and chemical nature from the side of the CEL and the AEL. When the system is in the underlimiting mode (*i* < *i*_lim_), the only charge carriers are salt ions. In the sodium chloride solution, the chloride ion is transported from the left solution into the CEL, and the sodium ion is transported from the right solution into the AEL.

Due to the nature of the bilayer membrane, the concentration of ions is depleted on both sides of the membrane. In the case of a conventional isotropic membrane, the concentration of ions is depleted on one side and is enriched on the other.

The chloride anions penetrate the cation-exchange film as co-ions; hence, their concentration in the CEL will be substantially lower than the solution; usually, it is at least an order of magnitude lower. As a result, the concentration of chloride ions at the CEL/AEL interface reaches zero at currents much smaller than in the anion-exchange membrane-substrate. Thus, the control of the electrodiffusion kinetics passes from the diffusion layer in the solution to the thin cation-exchange layer.

Neglecting the water splitting reaction at the cation-exchanger/anion-exchanger interface, the limiting current density for a bipolar membrane can be expressed as the sum of electrodiffusion limiting currents in each of the layers [85]:

(10) $$\hat{i}\frac{c_{l}^{2}}{\beta_{c}}{\frac{c_{r}^{2}}{\beta_{a}}}_{lim},$$

where:

(11) $$\beta_{c}=\frac{\frac{d_{c}Q_{c}}{D_{c}}}{\frac{d_{a}Q_{a}}{D_{a}}+\frac{d_{c}Q_{c}}{D_{c}}},\beta_{a}=1-\beta_{c},$$

The reduced current density (which has the dimension of concentration in a square) in Equation (10) is related to the current density, which has the dimension A/m^2^, by the following relationship (for a binary 1-1 electrolyte):

(12) $$\hat{i}=\frac{i}{F}\left(\frac{d_{a}Q_{a}}{D_{a}}+\frac{d_{c}Q_{c}}{D_{c}}\right),$$

In the case of a bilayer membrane, the thickness of the cation-exchange layer is much less than the thickness of the substrate membrane; therefore, we will consider β_c_ → 0, then substituting Equations (11) and (12) into Equation (10), we obtain the full expression for the limiting current of the bilayer membrane with one thin layer:

(13) $$i{\left(\frac{c_{l}^{2}D_{c}}{d_{c}Q_{c}}+\frac{c_{r}^{2}\left(D_{c}+D_{a}\right)}{d_{a}Q_{a}D_{c}+d_{c}Q_{c}D_{a}}\right)}_{lim}.$$

Because we consider that, the AEL is thicker than the CEL (d_a_ ≫ d_c_), the second term on the right side of the Equation (13) becomes at least an order of magnitude larger than the first one. Then, only the first term will have a key contribution to the limiting current value. Therefore, for a bilayer membrane with a thin cation-exchange layer, we can write:

(14) $$i{\frac{FD_{c}c_{l}^{2}}{d_{c}Q_{c}}}_{lim}.$$

For the membrane with the thin anion-exchange layer, the notation in the Equation (14) should be changed to *c_r_*, *D_a_*, *d_a_* and *Q_a_*, while the above reasoning will also be valid.

In the general case, when a direct current flows in a solution, concentration polarization is observed and the ion concentrations at the membrane/solution interface depend on the current density and cannot be specified exactly. Thus, the properties of the bilayer membrane become dependent on convective-diffusion processes in the external solution.

As an approximation, let us assume that Nernst diffusion layers are formed at the membrane/solution interface (linear concentration profile). Then, the concentration of the binary 1-1 electrolyte at the membrane surface at a certain current *i* will be equal to:

(15) $$c_{l}=c_{l}^{0}-\frac{1}{2}\frac{i\delta }{FD_{\mathrm{sol}}}.$$

Note that, in the general case, the current (*i*) in Equation (15) is not equal to the limiting current that is observed experimentally (*i*_lim_ in Equation (14)), since the limiting current in the bilayer membrane is determined by the concentration profiles of ions inside the cation-exchange layer. Substituting Equation (15) into Equation (14), we get:

(16) $$\frac{i_{lim}}{\frac{FD_{c}{\left(c_{l}^{0}\right)}^{2}}{d_{c}Q_{c}}\frac{i\delta }{FD_{\mathrm{sol}}c_{l}^{0}}{\left(\frac{i\delta }{2FD_{\mathrm{sol}}c_{l}^{0}}\right)}^{2}}.$$

Let’s introduce the notation:

(17) $$i_{lim}^{m{\frac{FD_{c}{\left(c_{l}^{0}\right)}^{2}}{d_{c}Q_{c}}}_{lim}^{d\frac{2FD_{sol}c_{l}^{0}}{\delta }}},$$

where $i_{lim}^{m}$ is the limiting current determined by a thin cation-exchange layer, $i_{lim}^{d}$ is the limiting current determined by the electrodiffusion of ions in the solution in the absence of a film on the surface of the substrate membrane. Equation (17) for $i_{lim}^{m}$ differs from Equation (14) in that there is no dependence on the electrolyte concentration at the membrane/solution interface, and the value of the limiting current is determined only by the properties of the cation-exchange layer and the concentration of ions in the depth of the solution.

After substituting Equation (17) into Equation (16) and for *i* = *i*_lim_ we obtain:

(18) $$\frac{i_{lim}}{i_{lim}^{m}=\left(1-{\frac{i_{lim}}{i_{lim}^{d}}}^{2}\right)}.$$

The solution to the resulting expression will be:

(19) $$i{\left(\frac{2\alpha +1-\sqrt{4\alpha +1}}{2\alpha }\right)}_{{lim}_{lim}}^{d},$$

where *α* is the ratio of the values of the limiting currents in the membrane and in the diffusion layer:

(20) $$\alpha =\frac{i_{lim}^{m}}{i_{lim}^{d}=\frac{1}{2}\frac{\delta D_{c}^{*}c_{l}^{0}}{d_{c}D_{sol}Q_{c}}}.$$

where $D_{c}^{*}$ is some effective diffusion coefficient in a thin layer of a bilayer membrane.

If α takes values less than unity, then we should talk about the limiting current controlled by the membrane. If α takes values greater than unity, then it should be said that the determining mechanism for the occurrence of the limiting state is the electrodiffusion of the electrolyte in the diffusion layer.

**Figure 21:**
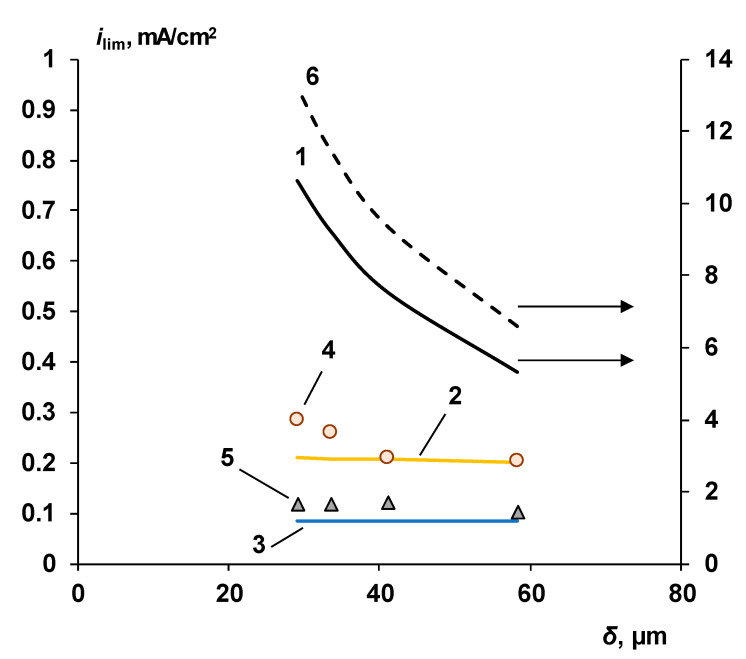
Dependence of the limiting current density on the diffusion boundary layer thickness. 1—calculated using Equation (17), 2—calculated using Equation (19) for BM-a-10, 3—calculated using Equation (19) for BM-a-30, 4—experimental data for BM-a-10, 5—experimental data for BM-a-30, 6—calculation using the modified Levich’s equation [86].

As shown in Section 3.1, for membranes with a thin cation-exchange layer, there is an explicit dependence of the transport number of chloride ions, not only on the current density, but also on the thickness of the cation-exchange layer. Earlier, we showed the dependence of the limiting current on the thickness of the cation-exchange layer, calculated theoretically and found experimentally [71]. In this case, for BM-a bilayer membranes, there is practically no dependence of the limiting current on hydrodynamics (the thickness of the depleted diffusion layer), as evidenced by the fact that the membrane samples studied using a rotating membrane disk [77] do not obey the Levich equation (Figure 21). The limiting current values calculated using Equation (17) are also much larger than the experimentally observed values.

The use of Equation (19) gives satisfactory agreement between the experiment and the calculated values, provided that the value of $D_{c}^{*}$ is of the same order of magnitude as the diffusion coefficient of the electrolyte in solution (*D*_sol_). As a rule, the diffusion coefficients of ions in the membrane are taken to be an order of magnitude lower than in the solution. It should be noted that the obtained equations do not consider the real physical state of both the cation-exchange layer and the substrate membrane. Violations of the integrity of the cation-exchange film, uneven thickness of the film on the surface of the substrate membrane, a large fraction of the intergel solution through which diffusion of co-ions proceeds—all these factors can lead to “overestimated” diffusion coefficients of chloride ions through the cation-exchange layer.

If $\frac{D_{c}^{}}{D_{sol}}$ taken equal to about 0.1 (from the results shown in Figure 6, as well as from independent experiments [87], it was established that the diffusion coefficient of the chloride ion in a cation-exchange layer 30 microns thick is 9.8·10^−7^–1.4·10^−6^ cm^2^/s), then the values of the limiting currents for the studied membranes will be 0.01 mA/cm^2^ for the BM-a-10 membrane and 0.004 mA/cm^2^ for the BM-a-30 membrane. Note that in real experiments, such values of limiting currents in solutions of corresponding concentrations were not encountered. The reason for such a significant discrepancy between the observed value of the co-ion diffusion coefficient and the limiting current has not been established. In this regard, in Equation (20), the value of the diffusion coefficient ($D_{c}^{}$) is replaced by some effective diffusion coefficient ($D_{c}^{*}$).

From the obtained Equation (20), it is possible to estimate the thickness of the cation-exchange layer at which the limiting current in a system with a bilayer membrane is controlled by diffusion, and at which values of the limiting current in the solution and in the film will coincide. For this, the α value must be greater than unity:

(21) $$\frac{1}{2}\frac{\delta D_{c}c_{l}^{0}}{d_{c}D_{sol}Q_{c}}\geq 1.$$

If $\frac{c_{l}^{0}}{Q_{c}}\approx 0.01$ (for $c_{l}^{0}$ = 0.01 M); $\frac{D_{c}}{D_{sol}}\approx 0.1$ we obtain:

(22) $$d_{c}\geq \frac{\delta }{2}\times 0.001.$$

Thus, the limiting current on a BM-a series bilayer membrane is practically independent of the hydrodynamic conditions in the depleted diffusion layer at a cation-exchange layer thickness of about 25 nm (provided that δ = 50 microns, $c_{l}^{0}$ = 0.01 M). Even if we take into account the aforementioned condition that $\frac{D_{c}^{*}}{D_{sol}}\approx 1$, then the CEL thickness should be less than 250 nm thick to change the nature of the limiting current from membrane-controlled to be diffusion-controlled. Since the thickness of the diffusion layer depends on hydrodynamic conditions and, as a rule, is tens of micrometers, the only effective way to increase the limiting current on the bilayer membrane is to increase the concentration of the external solution.

#### The Co-Ion Leakage through Membranes BM-ac

In the case of BM-ac series membranes with KF-1, the calculations made above cannot be applied, since the structure of such a membrane is much more complicated; in addition, due to the high catalytic activity of KF-1, the flow of the water splitting reaction cannot be ignored even at low currents.

The apparent reason for the appearance of a sufficiently high limiting current (higher than for the BM-a series membranes) is the loss of membrane selectivity. The decrease in the selectivity to hydrogen and hydroxyl ions observed for the BM-ac membranes with 0.2 and 2 mg/cm^2^ of catalyst compared with BM-a membranes (Figure 11) is associated with the complex structure of its cation-exchange layer. The cation-exchange layer of the BM-ac contains microscopic catalyst particles. The presence of mechanical dispersion in the cation-exchange film can lead to the appearance of mechanical irregularities in the cation-exchanger layer, a decrease in the thickness of the film over the catalyst pellets. The presence of large catalyst particles is also important. Those particles may serve as paths for the non-selective transfer of anions. It is known that phosphoric acid ionogenic groups, on the one hand, have high catalytic activity in the water splitting reaction, and on the other, lose their selectivity (the ability to retain anions) when they enter the acid medium [88], due to the protonation of phosphoric acid groups at low pH values, according to the reactions:

(23) $$–{\mathrm{PO}}_{3}^{2-}\;+\;H_{2}O\;\overset{\;\;\;\;\;\;\;\;\;\;k_{1}=10^{1}÷10^{3}s^{-1}\;\;\;}{\to }\;\underset{\;k_{-1}=10^{8}÷10^{10}L/\left(\mathrm{mols}\right)\;}{\leftarrow }\;–{\mathrm{PO}}_{3}H^{-}\;+\;{\mathrm{OH}}^{-},$$

(24) $$–{\mathrm{PO}}_{3}H^{-}\;+\;H_{2}O\;\overset{\;\;k_{1}=5\cdot 10^{-3}÷5\cdot 10^{-1}s^{-1}\;\;\;}{\to }\;\underset{\;k_{-1}=10^{8}÷10^{10}L/\left(\mathrm{mol}\cdot s\right)\;}{\leftarrow }\;–{\mathrm{PO}}_{3}H_{2}\;+\;{\mathrm{OH}}^{-}.$$

Because of the Reactions (23) and (24), the selectivity of the cation-exchange layer decreases and a non-selective transfer of chloride ions from the acid chamber to the alkaline chamber occurs. At the same time, the thick membrane-substrate prevents the transfer of sodium ions.

In the case of a membrane containing 6 mg/cm^2^ of catalyst, the main source of non-selective transfer is the physical inhomogeneity of the cation-exchange film. About a third of all particles have a size larger than the thickness of the cation-exchange layer. This leads to its breakthrough by large particles or catalyst agglomerates, the emergence of new pathways for the electrodiffusion transport of chloride ions directly to the surface of the anion-exchange membrane and through it.

### 4.2. The Water Splitting Reaction Kinetics

The resistance of the bipolar region in Equation (8) is [79]:

(25) $$R_{b}=\frac{RT{\overset{-}{t}}_{H^{+}}}{F^{2}{\left(c_{H^{+}}\right)}_{x=0}\sqrt{D_{H^{+}}\chi }}.$$

Taking the logarithm from both sides of the Equation (25) leads to a linear dependence of lg*R_b_* on lg*χ*:

(26) $$\mathrm{lg}R_{b}=-\frac{1}{2}\mathrm{lg}\chi +\mathrm{lg}\left(\frac{RT{\overset{-}{t}}_{H^{+}}}{F^{2}{\left(c_{H^{+}}\right)}_{x=0}\sqrt{D_{H^{+}}}}\right),$$

with the slope equal to ½.

**Figure 22:**
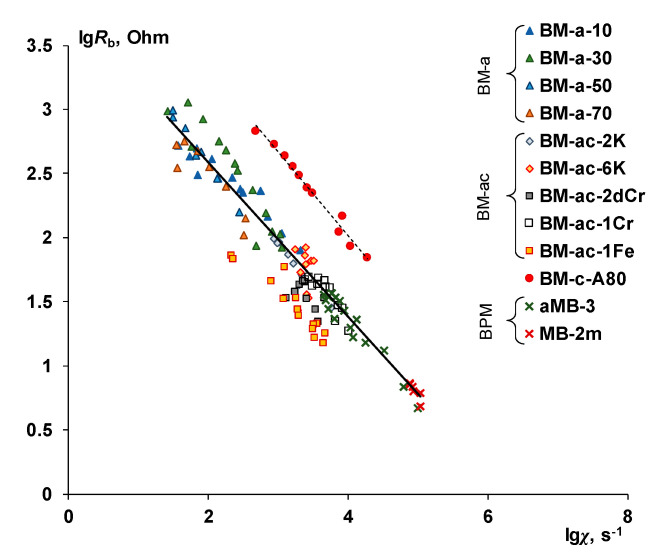
Logarithmic dependence of bipolar region resistance on non-equilibrium effective water-splitting rate constant for bilayer and bipolar membranes. Data for aMB-3 membrane were acquired by the authors, data for MB-2m were processed from [61], other points are from data presented in Section 3.

We obtained the values of the non-equilibrium effective rate constants of the water splitting reaction (*χ*) and the reaction layer’s resistance (*R_b_*), depending on the current density and the nature of the membrane by numerical fitting of the impedance parameters, according to Scheme 1 to the spectra of the electrochemical impedance of bilayer membranes presented in Section 3. The results are shown in Figure 22.

The results obtained both for bilayer membranes with and without a catalyst for the water splitting reaction, as well as for bipolar membranes aMB-3 and MB-2m [61], generally fall on one straight line in logarithmic coordinates, but the slope of this straight line is 0.60 ± 0.05, which is slightly more than the expected value of ½.

To compare the efficiency of different catalysts with each other, authors of [89] suggested using the value of the effective water-splitting rate constant in the absence of an external electric field (*k*_Σ_). The *k*_Σ_ is independent on current and shows the water splitting efficiency under thermodynamic equilibrium, while *χ* is dependent on current. The *k*_Σ_ can be found using the experimentally obtained dependence of the partial current by the water splitting products on the magnitude of the overvoltage of the bipolar region (a detailed derivation of the equation can be found in [71,89]):

(27) $$i_{H^{+},{\mathrm{OH}}^{-}}=\frac{k_{\Sigma }}{\beta }\varepsilon \varepsilon_{0}e^{\beta E_{m}}-i_{H_{2}O}^{r}.$$

In Equation (27) *E_m_* is a function of bipolar region overvoltage ($\eta_{b}$) [89].

Based on the obtained experimental data, using the Equation (27), the effective water splitting rate constant (*k*_Σ_) was calculated by minimizing the residual dispersion of the experimental points with respect to the calculated curve.

The calculated values of the effective rate constants of the water splitting reaction for the studied membranes are shown in Table 6.

As can be seen from the table, for the BM-ac series membranes with the KF-1 catalyst, regardless of the amount of catalyst, the value of the effective constant *k_Σ_* changes insignificantly. Despite the apparent discrepancy with the voltammetry data, where, with an increase in the amount of catalyst, the voltage drop on the membrane decreases (Figure 9), this effect is easily explained by the dependence of the transport numbers on the amount of catalyst (Figure 11). The decrease in selectivity caused by the physical inhomogeneity of the cation-exchange layer leads to a local decrease in the electric field strength in the space-charge region and to a decrease in the effective generating area of the bipolar contact.

In the case of electrodeposited catalysts of chromium (III) hydroxide, it can be concluded that only a small fraction of the area of the heterogeneous catalyst is active in the water splitting reaction, namely, that part that is localized in the space charge region, where the reaction takes place. This is evidenced by the decrease in the effective constant kΣ as the deposition time increases. The part of the catalyst that is outside the space charge region does not participate in catalysis. Since the thickness of the space charge region is about 1–5 nm [76,90], only particles deposited directly on the bipolar boundary, even in small amounts, have a significant effect on the voltage drop across the membrane. This conclusion coincides with the conclusions made in [90]; however, in our work, it is shown experimentally.

### 4.3. Future Perspectives for Bilayer Membranes

The strong dependence of the current efficiency of hydrogen and hydroxyl ions on the asymmetric bipolar membrane on the current density makes it possible to vary the ratio of salt ions and water-splitting products fluxes by changing the current.

From measurements of the effective transport numbers of ions across bilayer membranes, they can find application in three different modes.

The first one, the “selective” mode, is the separation of monovalent and polyvalent ions [41]. This mode is realized when the current is less than the limiting current and salt ions are charge carriers.

The second mode is the “mixed” mode, when salt ions are transported while the water splitting reaction proceeds. In this mode, the water splitting reaction current is in the range 0 < *i_w_* < 0.9*i_s_*.

The third mode is controlled by the kinetics of the water splitting reaction, and here the bilayer membrane performs the same functions as the bipolar membrane. In this mode, the main charge carriers are hydrogen and hydroxyl ions and the water splitting reaction current is *i_w_*> 0.9*i_s_*.

**Figure 23:**
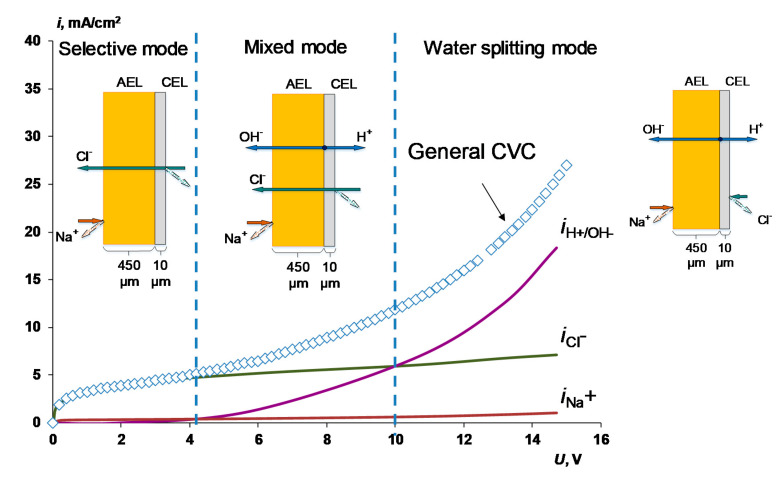
General and partial by ions current-voltage curves of BM-a-30 membrane measured in 0.1 M NaCl. Schematically, the main processes in each mode are shown as well.

The different modes are shown schematically in Figure 23.

The ability to regulate the transport numbers of salt and hydrogen and hydroxyl ions by changing the current density makes it possible to conduct a controlled process of desalting solutions of electrolytes with simultaneous pH adjustment.

## 5. Conclusions

In this work, we studied a complex of electrochemical characteristics of bilayer membranes that differ in the structure and properties of the layers (a thin modifying layer and a substrate membrane). The obtained characteristics were compared between both the studied bilayer membranes and well-known commercial bipolar membranes. The latter can be considered as an extreme case of bilayer membranes, in which the layer thicknesses are of the same order of magnitude.

The results obtained can be classified into several categories:

1. When a thin layer of an ion-polymer is deposited onto a membrane-substrate with a matrix charge opposite to that of a matrix of the membrane-substrate, an abrupt change in the electrochemical behavior of the resulting bilayer membrane occurs. There is a sharp decrease in the value of the limiting current, which is caused by a change in the nature of the limiting current in electromembrane systems with bilayer membranes. The limiting current in a system with a bilayer membrane is determined not by diffusion restrictions in the solution, but by the electrodiffusion of co-ions (relative to the charge of the thin layer matrix) in the ion-polymer film deposited on the substrate membrane. Previously, we observed such changes only for membranes with a thin cation-exchange layer; however, the same effects are also caused by the deposition of a thin layer of anion-exchanger on the surface of the cation-exchange membrane-substrate. The second important feature is a complete transition to the “generation mode” in an overlimiting state, while for the original Ralex membranes, a mixing of the modes of electroconvection and generation of H^+^/OH^−^ ions is noted [91].
2. It is shown that irrespective of the nature of the catalyst or its absence, as well as the thickness of the layers forming the membrane, the process of water splitting in the bipolar region gives a linear relationship between lg*R_b_* and lgχ. At the same time, for all studied membranes, a slight deviation of the slope of dependence from the theoretical value of ½ is observed, which may indicate an incomplete correspondence of modern theoretical concepts of the electrochemical spectrum of the impedance of a bipolar (bilayer) membrane as the Gerischer impedance.
3. Water splitting catalysts of different chemical nature have different effects on the transport properties of bilayer membranes. The use of non-conductive materials (iron (III) and chromium (III) hydroxides) does not lead to a significant flux of salt ions, compared with the original bilayer membrane without a catalyst, which, together with the high catalytic activity of these substances, allows the use of bilayer membranes instead of bipolar in solutions, with a concentration of up to 0.5 M. At the same time, the use of a phosphoric acid ion-polymer catalyst leads to voltage/selectivity trade-off: on the one hand, the potential drop across membrane significantly decreases, on the other, the limiting current increases.
4. Under certain conditions for bilayer membranes, it is possible to achieve a significant transfer of salt ions. These effects are especially pronounced in membrane samples with a phosphoric acid catalyst for the water splitting reaction. The significant contribution of the transport of salt ions, simultaneously with the intensive water splitting, makes it possible to use such membranes in processes that simultaneously require the transfer of salt ions and a change in pH. For example, when processing fruit juices, preparing water for heat power engineering, and carrying out the kinetic separation of inorganic and organic electrolytes. Studying the functioning of bilayer membranes in mixed-mode processes seems to be the most interesting in the future.

## Figures and Tables

**Table 1:** Studied membranes nomenclature.

Membrane Name	Series	AEL	CEL	Thickness of CEL/AEL, Microns	Catalyst	Mass of the Catalyst, mg/cm^2^ Deposition Time, min
BM-a-10	BM-a	Ralex AMH-PES	sulfonated polytetrafluoroethylene	10	-	-
BM-a-30				30		
BM-a-50				50		
BM-a-70				70		
BM-a-SPEEK			sulfonated poly(ether ether)ketone	30		
BM-a-SPS			sulfonated polystyrene	30		
BM-c-A80	BM-c	Ralex CM-PES	Copolymer*	80		
BM-ac-0.2K	BM-ac	Ralex AMH-PES	sulfonated polytetrafluoroethylene	30	KF-1 (microdispersion)	0.2
BM-ac-2K						2
BM-ac-6K						6
BM-ac-2dCr					Cr(OH)_3_ (microdispersion)	2
BM-ac-2dFe					Fe(OH)_3_ (microdispersion)	2
BM-ac-1Cr					Cr(OH)_3_ (electrodeposition)	1 **
BM-ac-2Cr						2 **
BM-ac-10Cr						10 **
BM-ac-1Fe					Fe(OH)_3_ (electrodeposition)	1 **
BM-ac-solFe					Fe(OH)3 (sol)	-

* See Section 2.1.2. ** Here the numbers indicate the deposition time.

**Table 2:** Transport numbers, water-splitting potential and limiting current value for BM-a membranes in the acid-alkali system with 0.01 M concentrations.

Membrane	BM-a-10	BM-a-30	BM-a-50	BM-a-70
CEL thickness, μm	10	30	50	70
$t_{{\mathrm{Na}}^{+}}^{m}$	0.05 ± 0.03	0.05 ± 0.03	0.04 ± 0.02	0.02 ± 0.02
$t_{{\mathrm{Cl}}^{-}}^{m}$	0.14 ± 0.03	0.09 ± 0.02	0.06 ± 0.01	0.05 ± 0.01
$t_{H^{+}/{\mathrm{OH}}^{-}}^{m}$	0.81 ± 0.05	0.86 ± 0.04	0.89 ± 0.03	0.91 ± 0.03
Δ*U*_diss,_ V	2.5	4.1	3.3	2.9
*i_lim_*, mA/cm^2^	0.38	0.05	-	-

**Table 3:** Effective water-splitting rate constant for bilayer membranes with different chemical nature of the CEL.

Cation-Exchange Layer	Current Density *i*, mA/cm^2^		
	2.0	2.5	3.0
SPTFE	3.9	5.0	5.4
SPEEK	3.4	4.2	5.1
SPS	2.1	1.7	1.9

**Table 4:** Transport numbers, water-splitting potential and limiting current value for BM-ac with KF-1 catalyst membranes in the acid-alkali system with 0.01 M concentrations.

Membrane	BM-ac-02K	BM-ac-2K	BM-ac-6K
Amount of catalyst, mg/cm^2^	0.2	2	6
$t_{{\mathrm{Na}}^{+}}^{m}$	0.05 ± 0.01	0.05 ± 0.01	0.05 ± 0.01
$t_{{\mathrm{Cl}}^{-}}^{m}$	0.09 ± 0.02	0.13 ± 0.02	0.21 ± 0.03
$t_{H^{+}/{\mathrm{OH}}^{-}}^{m}$	0.86 ± 0.02	0.81 ± 0.02	0.74 ± 0.05
Δ*U*_diss,_ V	1.6	0.9	0.75
*i_lim_*, mA/cm^2^	-	-	0.25

**Table 5:** Transport numbers, water-splitting potential and limiting current value for BM-a-30 and BM-ac-2K membranes in the acid-alkali system with various concentrations.

Membrane	BM-a-30			BM-ac-2K		
Concentration, mol/L	0.01	0.1	0.5	0.01	0.1	0.5
$t_{{\mathrm{Na}}^{+}}^{m}$	0.05 ± 0.03	0.02 ± 0.02	0.03 ± 0.02	0.05 ± 0.03	0.01 ± 0.02	0.01 ± 0.03
$t_{{\mathrm{Cl}}^{-}}^{m}$	0.09 ± 0.02	0.17 ± 0.04	0.15 ± 0.05	0.13 ± 0.04	0.08 ± 0.04	0.39 ± 0.04
$t_{H^{+}/{\mathrm{OH}}^{-}}^{m}$	0.86 ± 0.04	0.81 ± 0.04	0.82 ± 0.07	0.82 ± 0.04	0.91 ± 0.04	0.60 ± 0.04
Δ*U*_diss,_ V	3.7	5.0–5.5	9–10	1.2	1.8	2.3
*i_lim_*, mA/cm^2^	0.05	0.6	3.1	-	1.8	15

**Table 6:** Effective rate constants of the water splitting reaction for the studied bilayer membranes.

Membrane	BM-a-10	BM-a-30	BM-a-50	BM-a-70	BM-ac-0.2K	BM-ac-2K	BM-ac-6K	BM-ac-2dCr	BM-ac-1Fe	BM-ac-1Cr	BM-ac-2Cr	BM-ac-10Cr
*k_Σ_*, s^−1^	3.29	2.68	4.47	6.14	16.9	18.3	19.0	38.3	9.72	92.2	38.3	4.30

## Supplementary Materials

The following are available online at https://www.mdpi.com/2077-0375/10/11/346/s1, Figure S1: Size dispersion of catalyst particles.

## Abbreviations

Subscripts *c* and *a* refer to the parameters of the cation-exchange layer and the anion-exchange layer respectively.

AEL	anion-exchange layer	
CEL	cation-exchange layer	
BM-a	bilayer membranes with thin cation-exchange layer and without a water splitting catalyst	
BM-ac	bilayer membranes with thin cation-exchange layer and with a water splitting catalyst	
SPEEK	sulfonated poly (ether ether) ketone	
SPS	sulfonated polystyrene	
SPTFE	sulfonated polytetrafluoroethylene	
Parameter	Dimensions	Description
*P_j_*	L/s	permeability coefficient of acid or alkali
Δc_i_	mol/L	difference in concentration of acid or alkali in the depth of the solution on both sides of the membrane
*S*	cm^2^	membrane area
$c_{{\mathrm{Na}}^{+}/{\mathrm{Cl}}^{-},}\cdot c_{{\mathrm{Na}}^{+}/C1^{-}}^{0}$	mol/L	concentrations of sodium and chloride ions in the solution, at the outlet the cell, and in feed solution
$T_{{\mathrm{Na}}^{+}/{\mathrm{Cl}}^{-}}$		effective transport number of sodium or chloride ion
*T*_w_		effective transport number of H^+^ and OH^−^ ions
*W*	L/s	volume flow rate of a solution
*i*	mA/cm^2^	current density
*i*_lim_	mA/cm^2^	limiting current density
$R_{b},R_{0},R_{\infty }$	Ohm·cm^2^	Resistances of the bipolar region, ohmic resistance and total resistance of the bilayer membrane
$\eta_{b}$	V	overvoltage of the bipolar region
ω	rad/s	angular frequency
*χ*	1/s	non-equilibrium effective rate constant of the water splitting reaction
*c_l_, c_r_*	mol/L	concentration of electrolyte on the membrane/solution boundary to the left and to the right of the membrane
$c_{l}^{0},c_{r}^{0}$	mol/L	bulk concentration of the electrolyte on the left side of the membrane
$\beta_{c^{\prime }}\beta_{a}$		characteristic thicknesses of the CEL and AEL layers respectively
*d_c_, d_a_*	m	thickness of the CEL and AEL layers respectively
*Q_c_, Q_a_*	mol/m^3^	ion-exchange capacity of the CEL and AEL layers respectively
*D_c_, D_a_*	m^2^/s	co-ion diffusion coefficient inside the CEL and AEL layers respectively
*D*_sol_	m^2^/s	salt diffusion coefficient in the solution
δ	m	diffusion boundary layer thickness
$i_{\lim}^{m}$	mA/cm^2^	limiting current determined by a thin cation-exchange layer
$i_{\lim}^{d}$	mA/cm^2^	limiting current determined by the electrodiffusion of ions in the solution in the absence of a film on the surface of the substrate membrane
*α*		ratio of the values of the limiting currents in the membrane and in the diffusion layer
${\bar{t}}_{H^{+}}$		electromigration transport number of hydrogen ion in the CEL
${\left(c_{H^{+}}\right)}_{x=0}$	mol/m^3^	concentration of hydrogen ion at the CEL/AEL interface
$D_{H^{+}}$	m^2^/s	hydrogen ion diffusion coefficient inside the CEL
$i_{H^{+},{\mathrm{OH}}^{-}}$	mA/cm^2^	current carried by the hydrogen and hydroxyl ions
$i_{H_{2}O}^{r}$	mA/cm^2^	current of the water recombination reaction in the absence of an external electric field
*β*	m/V	empirical parameter, which can be related to a characteristic length
$k_{\Sigma }=c_{H_{2}O}\left(k_{20}+k_{40}\right)x$	1/s	total effective rate constant of the pseudomonomolecular water splitting reaction in the space charge region in the absence of an electric field
*E*_m_	V/m	electric field strength at the CEL/AEL interface
ε		relative permittivity of the space charge region
$\varepsilon_{0}$	F/m	absolute dielectric constant of vacuum
*R*	J/(mol·K)	universal gas constant
*T*	K	temperature
*F*	A·s/mol	Faraday constant

## References

[1] Bazinet L., Lamarche F., Ippersiel D. (1998). Bipolar-membrane electrodialysis: Applications of electrodialysis in the food industry. Trends Food Sci. Technol..

[2] Strathmann H. (2010). Electrodialysis, a mature technology with a multitude of new applications. Desalination.

[3] Xu T. (2002). Electrodialysis processes with bipolar membranes (EDBM) in environmental protection—A review. Resour. Conserv. Recycl..

[4] Gutiérrez L.-F., Bazinet L., Hamoudi S., Belkacemi K. (2013). Production of lactobionic acid by means of a process comprising the catalytic oxidation of lactose and bipolar membrane electrodialysis. Sep. Purif. Technol..

[5] Jaroszek H., Dydo P. (2016). Ion-exchange membranes in chemical synthesis—A review. Open Chem..

[6] Pärnamäe R., Mareev S., Nikonenko V., Melnikov S., Sheldeshov N., Zabolotskii V., Hamelers H.V.M., Tedesco M. (2020). Bipolar membranes: A review on principles, latest developments, and applications. J. Memb. Sci..

[7] Readi O.M.K., Gironès M., Wiratha W., Nijmeijer K. (2013). On the isolation of single basic amino acids with electrodialysis for the production of biobased chemicals. Ind. Eng. Chem. Res..

[8] Nir O., Sengpiel R.G., Wessling M. (2018). Closing the cycle: Phosphorus removal and recovery from diluted effluents using acid resistive membranes. Chem. Eng. J..

[9] Gurreri L., Tamburini A., Cipollina A., Micale G. (2020). Electrodialysis Applications in Wastewater Treatment for Environmental Protection and Resources Recovery: A Systematic Review on Progress and Perspectives. Membranes.

[10] Shi L., Hu Y., Xie S., Wu G., Hu Z., Zhan X. (2018). Recovery of nutrients and volatile fatty acids from pig manure hydrolysate using two-stage bipolar membrane electrodialysis. Chem. Eng. J..

[11] Patel A., Mungray A.A.K., Mungray A.A.K. (2020). Technologies for the recovery of nutrients, water and energy from human urine: A review. Chemosphere.

[12] Melnikova E.D., Pismenskaya N.D., Bazinet L., Mikhaylin S., Nikonenko V.V. (2018). Effect of ampholyte nature on current-voltage characteristic of anion-exchange membrane. Electrochim. Acta.

[13] Rybalkina O., Tsygurina K., Melnikova E., Mareev S., Moroz I., Nikonenko V., Pismenskaya N. (2019). Partial fluxes of phosphoric acid anions through anion-exchange membranes in the course of NaH2PO4 solution electrodialysis. Int. J. Mol. Sci..

[14] Melnikov S., Kolot D., Nosova E., Zabolotskiy V. (2018). Peculiarities of transport-structural parameters of ion-exchange membranes in solutions containing anions of carboxylic acids. J. Memb. Sci..

[15] Lee H.J., Hong M.K., Han S.D., Cho S.H., Moon S.H. (2009). Fouling of an anion exchange membrane in the electrodialysis desalination process in the presence of organic foulants. Desalination.

[16] Ghalloussi R., Garcia-Vasquez W., Chaabane L., Dammak L., Larchet C., Deabate S.V., Nevakshenova E., Nikonenko V.V., Grande D. (2013). Ageing of ion-exchange membranes in electrodialysis: A structural and physicochemical investigation. J. Memb. Sci..

[17] Qasim M., Badrelzaman M., Darwish N.N., Darwish N.A., Hilal N. (2019). Reverse osmosis desalination: A state-of-the-art review. Desalination.

[18] Pinnau I., Freeman B.D. (1999). Formation and Modification of Polymeric Membranes: Overview. Membrane Formation and Modification.

[19] Frilette V.J. (1956). Preparation and Characterization of Bipolar Ion Exchange Membranes. J. Phys. Chem..

[20] Leitz F.B. (1971). Cationic-Anionic Ion-Exchange Membrane. U.S. Patent.

[21] Kollsman P. (1966). Antipolarization Membrane Having Anionic and Cationic Areas. U.S. Patent.

[22] Antonov Y.A., Ponomarev M.I., Volkov S.A., Grebenyuk V.D. (1983). Production of alkali with simultaneous water desaltination in electrodialyzer with semi- bipolar membranes. Sov. J. Water Chem. Technol. (Engl. Transl. Khimiya Tekhnologiya Vody).

[23] Shendrik O.R., Ponomarev M.I., Grebenyuk V.D. (1986). Modification of monopolar ion exchange membranes for hydrogen and hydroxyl ions generation. J. Appl. Chem. USSR.

[24] Ramireza P., Manzanaresb J.A., Mafe S. (1991). Water dissociation effects in ion transport through anion exchange membranes with thin cationic exchange surface films. Berl. Bunsenges Physycal Chem..

[25] Bukhovets A.E., Eliseeva T.V., Oren Y. (2010). Fouling of anion-exchange membranes in electrodialysis of aromatic amino acid solution. J. Memb. Sci..

[26] Slouka Z., Senapati S., Yan Y., Chang H.C. (2013). Charge inversion, water splitting, and vortex suppression due to DNA sorption on ion-selective membranes and their ion-current signatures. Langmuir.

[27] Senapati S., Slouka Z., Shah S.S., Behura S.K., Shi Z., Stack M.S., Severson D.W., Chang H.C. (2014). An ion-exchange nanomembrane sensor for detection of nucleic acids using a surface charge inversion phenomenon. Biosens. Bioelectron..

[28] Balster J.H., Sumbharaju R., Srikantharajah S., Pünt I., Stamatialis D.F., Jordan V., Wessling M. (2007). Asymmetric bipolar membrane: A tool to improve product purity. J. Memb. Sci..

[29] Zabolotskii V.I., Sheldeshov N.V., Melnikov S.S. (2013). Effect of cation-exchange layer thickness on electrochemical and transport characteristics of bipolar membranes. J. Appl. Electrochem..

[30] Zabolotsky V., Utin S., Bespalov A., Strelkov V. (2015). Modification of asymmetric bipolar membranes by functionalized hyperbranched polymers and their investigation during pH correction of diluted electrolytes solutions by electrodialysis. J. Memb. Sci..

[31] Abdu S., Sricharoen K., Wong J.E., Muljadi E.S., Melin T., Wessling M. (2013). Catalytic polyelectrolyte multilayers at the bipolar membrane interface. Appl. Mater. Interfaces.

[32] Abdu S., Wessling M. (2014). Layer-by-Layer Modification of Cation Exchange Membranes Controls Ion Selectivity and Water Splitting. Appl. Mater. Interfaces.

[33] Rijnaarts T., Reurink D.M., Radmanesh F., de Vos W.M., Nijmeijer K. (2019). Layer-by-layer coatings on ion exchange membranes: Effect of multilayer charge and hydration on monovalent ion selectivities. J. Memb. Sci..

[34] Ahmad M., Tang C., Yang L., Yaroshchuk A., Bruening M.L. (2019). Layer-by-layer modification of aliphatic polyamide anion-exchange membranes to increase Cl^−^/SO_4_^2−^ selectivity. J. Memb. Sci..

[35] White N., Misovich M., Yaroshchuk A., Bruening M.L. (2015). Coating of Nafion membranes with polyelectrolyte multilayers to achieve high monovalent/divalent cation electrodialysis selectivities. ACS Appl. Mater. Interfaces.

[36] Decher G., Hong J.-D. (1991). Buildup of ultrathin multilayer films by a self-assembly process, 1 consecutive adsorption of anionic and cationic bipolar amphiphiles on charged surfaces. Makromol. Chemie. Macromol. Symp..

[37] Decher G. (1997). Fuzzy Nanoassemblies: Toward Layered Polymeric Multicomposites. Science.

[38] Decher G., Eckle M., Schmitt J., Struth B. (1998). Layer-by-layer assembled multicomposite films. Curr. Opin. Colloid Interface Sci..

[39] Yokoyama Y., Tanioka A., Miyasaka K. (1988). Enzyme immobilization in an asymmetric charged membrane. J. Memb. Sci..

[40] Fu R., Xu T., Yang W., Pan Z. (2003). Preparation of a mono-sheet bipolar membrane by simultaneous irradiation grafting polymerization of acrylic acid and chloromethylstyrene. J. Appl. Polym. Sci..

[41] Golubenko D., Yaroslavtsev A. (2020). Development of surface-sulfonated graft anion-exchange membranes with monovalent ion selectivity and antifouling properties for electromembrane processes. J. Memb. Sci..

[42] Wang M., Jia Y.X., Yao T.T., Wang K.K. (2013). The endowment of monovalent selectivity to cation exchange membrane by photo-induced covalent immobilization and self-crosslinking of chitosan. J. Memb. Sci..

[43] Zhang D., Wang Y. (2006). Synthesis and applications of one-dimensional nano-structured polyaniline: An overview. Mater. Sci. Eng. B.

[44] Xiao X., Shehzad M.A., Yasmin A., Ge Z., Liang X., Sheng F., Ji W., Ge X., Wu L., Xu T. (2020). Anion permselective membranes with chemically-bound carboxylic polymer layer for fast anion separation. J. Memb. Sci..

[45] Afsar N.U., Shehzad M.A., Irfan M., Emmanuel K., Sheng F., Xu T., Ren X., Ge L., Xu T. (2019). Cation exchange membrane integrated with cationic and anionic layers for selective ion separation via electrodialysis. Desalination.

[46] Yan Z., Zhu L., Li Y.C.C., Wycisk R.J.J., Pintauro P.N.N., Hickner M.A.A., Mallouk T.E.E. (2018). The balance of electric field and interfacial catalysis in promoting water dissociation in bipolar membranes. Energy Environ. Sci..

[47] Ariono D., Gede Wenten I. (2017). Surface modification of ion-exchange membranes: Methods, characteristics, and performance. J. Appl. Polym. Sci..

[48] Luo T., Abdu S., Wessling M. (2018). Selectivity of ion exchange membranes: A review. J. Memb. Sci..

[49] Afsar N.U., Ji W., Wu B., Shehzad M.A., Ge L., Xu T. (2019). SPPO-based cation exchange membranes with a positively charged layer for cation fractionation. Desalination.

[50] Ahmad M., Yaroshchuk A., Bruening M.L. (2020). Moderate pH changes alter the fluxes, selectivities and limiting currents in ion transport through polyelectrolyte multilayers deposited on membranes. J. Memb. Sci..

[51] Wang M., Liu X., Jia Y.X., Wang X.L. (2015). The improvement of comprehensive transport properties to heterogeneous cation exchange membrane by the covalent immobilization of polyethyleneimine. Sep. Purif. Technol..

[52] Pourcelly G. (2002). Electrodialysis with bipolar membranes: Principles, optimization, and applications. Russ. J. Electrochem..

[53] Huang C., Xu T., Zhang Y., Xue Y., Chen G. (2007). Application of electrodialysis to the production of organic acids: State-of-the-art and recent developments. J. Memb. Sci..

[54] Wilhelm F.G., Pünt I., Van Der Vegt N.F.A., Wessling M., Strathmann H. (2001). Optimisation strategies for the preparation of bipolar membranes with reduced salt ion leakage in acid—Base electrodialysis. J. Memb. Sci..

[55] Hao J., Yu L., Chen C., Li L., Jiang W. (2001). Preparation of bipolar membranes. J. Appl. Polym. Sci..

[56] Yang B., Zhang H. (2008). Preparation of a bipolar membrane by photografting polymerization. Front. Chem. China.

[57] Xue Y., Wang N., Huang C., Cheng Y., Xu T. (2009). Catalytic water dissociation at the intermediate layer of a bipolar membrane: The role of carboxylated Boltorn^®^ H30. J. Memb. Sci..

[58] Balster J.H., Srinkantharajah S., Sumbharaju R., Pünt I., Lammertink R.G.H.G.H., Stamatialis D.F.F., Wessling M. (2010). Tailoring the interface layer of the bipolar membrane. J. Memb. Sci..

[59] Rajesh A.M., Chakrabarty T., Prakash S., Shahi V.K. (2012). Effects of metal alkoxides on electro-assisted water dissociation across bipolar membranes. Electrochim. Acta.

[60] Manohar M., Shukla G., Pandey R.P., Shahi V.K. (2017). Efficient bipolar membrane with protein interfacial layer for optimal water splitting. J. Ind. Eng. Chem..

[61] Sheldeshov N.V., Zabolotskii V.I., Bespalov A.V., Kovalev N.V., Alpatova N.V., Akimova A.V., Mochalova T.V., Kovaleva V.I., Boyarishcheva A.Y. (2017). The influence of catalytic additives on electrochemical properties of bipolar membranes. Pet. Chem..

[62] Simons R. (1993). Preparation of a high performance bipolar membrane. J. Memb. Sci..

[63] Mischi E., Mantione D., Pastacaldi A., Botte L. (2005). Method for Making a Bipolar Membrane and Use of Resulting Bipolar Membrane. U.S. Patent.

[64] Peng F., Peng S., Huang C., Xu T. (2008). Modifying bipolar membranes with palygorskite and FeCl3. J. Memb. Sci..

[65] Li S.-D., Wang C.-C., Chen C.-Y. (2008). Preparation and characterization of a novel bipolar membrane by plasma-induced polymerization. J. Memb. Sci..

[66] Wang H., Ding F., Jin G., Li C., Meng H. (2017). Ultra-thin graphene oxide intermediate layer for bipolar membranes using atomizing spray assembly. Colloids Surfaces A Physicochem. Eng. Asp..

[67] Kishino M., Yuzuki K., Fukuta K. (2019). Bipolar Membrane. U.S. Patent Application.

[68] Cheng G., Zhao Y., Li W., Zhang J., Wang X., Dong C. (2019). Performance enhancement of bipolar membranes modified by Fe complex catalyst. J. Memb. Sci..

[69] Melnikov S.S., Shapovalova O.V., Sheldeshov N.V., Zabolotskii V.I. (2011). Effect of d-metal hydroxides on water dissociation in bipolar membranes. Pet. Chem..

[70] Melnikov S.S., Zabolotskii V.I., Sheldeshov N.V., Achoh A., Bondarev D. (2018). Catalysis of water splitting reaction in asymmetric bipolar membranes with different chemical composition of cation-exchange layer. Desalin. Water Treat..

[71] Melnikov S.S., Sheldeshov N.V., Zabolotskii V.I. (2018). Theoretical and experimental study of current-voltage characteristics of asymmetric bipolar membranes. Desalin. Water Treat..

[72] Kang M.-S., Tanioka A., Moon S.-H. (2002). Effects of Interface Hydrophilicity and Metallic Compounds on Water-Splitting Efficiency in Bipolar Membranes. Korean J. Chem. Eng..

[73] Kang M.S., Choi Y.J., Lee H.J., Moon S.H. (2004). Effects of inorganic substances on water splitting in ion-exchange membranes: I. Electrochemical characteristics of ion-exchange membranes coated with iron hydroxide/oxide and silica sol. J. Colloid Interface Sci..

[74] El Moussaoui R., Pourcelly G., Maeck M., Hurwitz H.D., Gavach C. (1994). Co-ion leakage through bipolar membranes Influence on I–V responses and water-splitting efficiency. J. Memb. Sci..

[75] Wilhelm F.G., van der Vegt N.F.A., Wessling M., Strathmann H. (2001). Chronopotentiometry for the advanced current–voltage characterisation of bipolar membranes. J. Electroanal. Chem..

[76] Hurwitz H.D., Dibiani R. (2004). Experimental and theoretical investigations of steady and transient states in systems of ion exchange bipolar membranes. J. Memb. Sci..

[77] Sharafan M.V., Zabolotsky V.I. (2014). Study of electric mass transfer peculiarities in electromembrane systems by the rotating membrane disk method. Desalination.

[78] Zabolotsky V.I., Achoh A.R., Lebedev K.A., Melnikov S.S. (2020). Permselectivity of bilayered ion-exchange membranes in ternary electrolyte. J. Memb. Sci..

[79] Kniaginicheva E., Pismenskaya N., Melnikov S., Belashova E., Sistat P., Cretin M., Nikonenko V. (2015). Water splitting at an anion-exchange membrane as studied by impedance spectroscopy. J. Memb. Sci..

[80] Pismenskaya N.D., Rybalkina O.A., Kozmai A.E., Tsygurina K.A., Melnikova E.D., Nikonenko V.V. (2020). Generation of H^+^ and OH^−^ ions in anion-exchange membrane/ampholyte-containing solution systems: A study using electrochemical impedance spectroscopy. J. Memb. Sci..

[81] Suendo V., Minagawa M., Tanioka A. (2002). Bipolar Interface Formation of Cationic Surfactant on the Surface of a Cation-Exchange Membrane: Current−Voltage Characteristics in Aqueous Electrolyte Solution. Langmuir.

[82] Alcaraz A., Holdik H., Ruffing T., Ramírez P., Mafé S. (1998). AC impedance spectra of bipolar membranes: An experimental study. J. Memb. Sci..

[83] Ashrafi A.M., Gupta N., Neděla D. (2017). An investigation through the validation of the electrochemical methods used for bipolar membranes characterization. J. Memb. Sci..

[84] Kovalchuk V.I., Zholkovskij E.K., Aksenenko E.V., Gonzalez-Caballero F., Dukhin S.S. (2006). Ionic transport across bipolar membrane and adjacent Nernst layers. J. Memb. Sci..

[85] Sonin A.A., Grossman G. (1972). Ion transport through layered ion exchange membranes. J. Phys. Chem..

[86] Zabolotskii V.I., Sharafan M.V., Sheldeshov N.V., Lovtsov E.G. (2008). Electric mass transport of sodium chloride through cation-exchange membrane MK-40 in dilute sodium chloride solutions: A rotating membrane disk study. Russ. J. Electrochem..

[87] Melnikov S., Shkirskaya S. (2019). Transport properties of bilayer and multilayer surface-modified ion-exchange membranes. J. Memb. Sci..

[88] Zabolotskii V.I., Sheldeshov N.V., Melnikov S.S. (2014). Heterogeneous bipolar membranes and their application in electrodialysis. Desalination.

[89] Umnov V.V., Shel’deshov N.V., Zabolotskii V.I. (1999). Current-voltage curve for the space charge region of a bipolar membrane. Russ. J. Electrochem..

[90] Mareev S.A., Evdochenko E., Wessling M., Kozaderova O.A., Niftaliev S.I., Pismenskaya N.D., Nikonenko V.V. (2020). A comprehensive mathematical model of water splitting in bipolar membranes: Impact of the spatial distribution of fixed charges and catalyst at bipolar junction. J. Memb. Sci..

[91] Belloň T., Polezhaev P., Vobecká L., Svoboda M., Slouka Z. (2019). Experimental observation of phenomena developing on ion-exchange systems during current-voltage curve measurement. J. Memb. Sci..

