# Erratum: Melnikov, S.; et al. Water Splitting and Transport of Ions in Electromembrane System with Bilayer Ion-Exchange Membrane. *Membranes* 2020, *10*, 346

**DOI:** 10.3390/membranes10120424

**Published:** 2020-12-15

**Authors:** Stanislav Melnikov, Denis Bondarev, Elena Nosova, Ekaterina Melnikova, Victor Zabolotskiy

**Affiliations:** Kuban State University, Stavropolskaya 149, 350040 Krasnodar, Russia; bondarew.denis1992@gmail.com (D.B.); firofran@mail.ru (E.N.); ekaterinabelashova23@gmail.com (E.M.); vizab@chem.kubsu.ru (V.Z.)

Due to an error during production, Equations (10), (13)–(20), (23), (24) were unreadable in the published paper [1]. The corrected Equations are provided below:

(10) $${\hat{i}}_{lim}=\frac{c_{l}^{2}}{\beta_{c}}+\frac{c_{r}^{2}}{\beta_{a}}$$

(13) $$i_{lim}=F{\left(\frac{c_{l}^{2}D_{c}}{d_{c}Q_{c}}+\frac{c_{r}^{2}\left(D_{c}+D_{a}\right)}{d_{a}Q_{a}D_{c}+d_{c}Q_{c}D_{a}}\right)}_{}$$

(14) $$i_{lim}={\frac{FD_{c}c_{l}^{2}}{d_{c}Q_{c}}}_{}.$$

(15) $$c_{l}=c_{l}^{0}-\frac{1}{2}\frac{i\delta }{FD_{\mathrm{sol}}}$$

(16) $$\frac{i_{lim}}{\frac{FD_{c}{\left(c_{l}^{0}\right)}^{{}^{2}}}{d_{c}Q_{c}}}=1-\frac{i\delta }{FD_{\mathrm{sol}}c_{l}^{0}}+{\left(\frac{i\delta }{2FD_{\mathrm{sol}}c_{l}^{0}}\right)}^{2}$$

(17) $$i_{lim}^{m}=\frac{FD_{c}{\left(c_{l}^{0}\right)}^{{}^{2}}}{d_{c}Q_{c}},i_{lim}^{d}=\frac{2FD_{\mathrm{sol}}c_{l}^{0}}{\delta }$$

(18) $$\frac{i_{lim}}{i_{lim}^{m}}={\left(1-\frac{i_{lim}}{i_{lim}^{d}}\right)}^{2}$$

(19) $$i_{lim}=\left(\frac{2\alpha +1-\sqrt{4\alpha +1}}{2\alpha }\right)i_{lim}^{d}{}_{}$$

(20) $$\alpha =\frac{i_{lim}^{m}}{i_{lim}^{d}}=\frac{1}{2}\frac{\delta D_{c}^{*}c_{l}^{0}}{d_{c}D_{\mathrm{sol}}Q_{c}}$$

(23) $$-{{\mathrm{PO}}_{3}}^{2-}\;+\;H_{2}O\;\overset{\;\;k_{1}=10^{1}÷10^{3}s^{-1}\;\;}{\underset{\;\;k_{-1}=10^{8}÷10^{10}L/(\mathrm{mol}\cdot s)\;\;}{\underset{\leftarrow }{\to }}}-{\mathrm{PO}}_{3}H^{-}+\;{\mathrm{OH}}^{-}$$

(24) $$-{\mathrm{PO}}_{3}H^{-}\;+\;H_{2}O\;\overset{\;\;k_{1}=5\cdot 10^{-3}÷5\cdot 10^{-1}s^{-1}\;\;}{\underset{\;\;k_{-1}=10^{8}÷10^{10}L/(\mathrm{mol}\cdot s)\;\;}{\underset{\leftarrow }{\to }}}-{\mathrm{PO}}_{3}H_{2}+\;{\mathrm{OH}}^{-}$$
