# Supplementary material for: Water Splitting and Transport of Ions in Electromembrane System with Bilayer Ion-Exchange Membrane
Source: Membranes (Basel). 2020 Nov 16;10(11):346. doi: 10.3390/membranes10110346 (PMC7697576; doi:10.3390/membranes10110346)
Supplement: Supplementary file 1 [file membranes-10-00346-s001.pdf]

membranes

MDPI

Supplementary materials

Water Splitting and Transport of Ions in Electromembrane System with Bilayer Ion-Exchange Membrane

Stanislav Melnikov *, Denis Bondarev, Elena Nosova, Ekaterina Melnikova and Victor Zabolotskiy

Kuban State University, Stavropolskaya 149, 350040 Krasnodar, Russia; bondarew.denis1992@gmail.com (D.B.); firofran@mail.ru (E.N.); ekaterinabelashova23@gmail.com (E.M.); vizab@chem.kubsu.ru (V.Z.)

* Correspondence: melnikov.stanislav@gmail.com

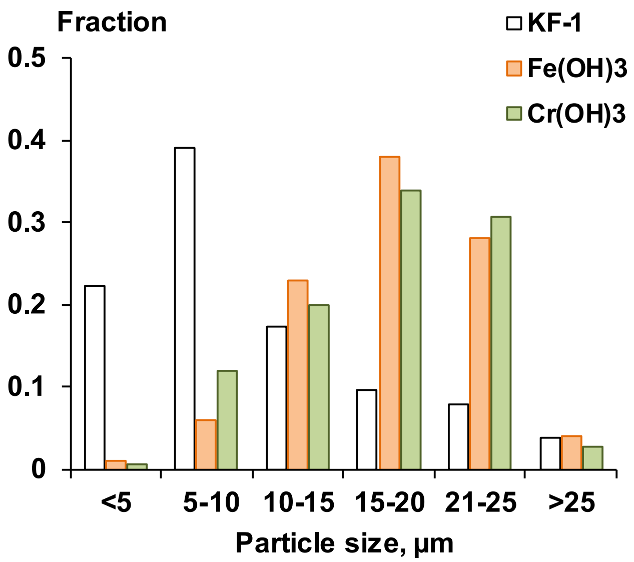

Figure S1. Size dispersion of catalyst particles
